# New Water-Soluble (Iminomethyl)benzenesulfonates Derived from Biogenic Amines for Potential Biological Applications

**DOI:** 10.3390/ma17020520

**Published:** 2024-01-22

**Authors:** Anna Kmieciak, Marek P. Krzemiński, Anastasiia Hodii, Damian Gorczyca, Aneta Jastrzębska

**Affiliations:** 1 Faculty of Chemistry, Nicolaus Copernicus University in Torun, 7 Gagarin Str., 87-100 Torun, Poland; mkrzem@umk.pl (M.P.K.); anastasiagodiy@gmail.com (A.H.); 2 Faculty of Medicine, Lazarski University, 43 Świeradowska Str., 02-662 Warsaw, Poland; damian.gorczyca@lazarski.pl; 3 LymeLab Pharma, Kochanowskiego 49A Str., 01-864 Warsaw, Poland

**Keywords:** biogenic amines, imine, sulfonate, antioxidant activity, inhibition of microorganism growth

## Abstract

In this paper, a highly efficient and straightforward method for synthesizing novel Schiff bases was developed by reacting selected biogenic amines with sodium 2-formylbenzene sulfonate and sodium 3-formylbenzene sulfonate. ^1^H and ^13^C NMR, IR spectroscopy, and high-resolution mass spectrometry were used to characterize the new compounds. The main advantages of the proposed procedure include simple reagents and reactions carried out in water or methanol and at room temperature, which reduces time and energy. Moreover, it was shown that the obtained water-soluble Schiff bases are stable in aqueous solution for at least seven days. Additionally, the antioxidant and antimicrobial activity of synthesized Schiff bases were tested.

## 1. Introduction

Imines are compounds containing a C=N group in their structure substituted on the carbon and nitrogen atoms with alkyl and aryl substituents. If the substituent on the nitrogen atom is an aryl group or a sterically extended alkyl substituent, such imines are commonly called Schiff bases (SBs). The most universal and most straightforward method for synthesizing SBs is the condensation reaction of primary amines with aldehydes and ketones providing SBs, often called aldimines and ketimines, respectively. This reaction takes place via the nucleophilic addition mechanism. Initially, the reaction produces an unstable α-aminoalcohol intermediate, which eliminates water molecules, leading to the formation of an imine. This reaction is reversible and requires water removal using dehydrating agents or azeotropic distillation.

Currently, new catalytic methods of SB synthesis are being developed [1], using the oxidation of amines [2], and aerobic amine coupling has also been recently reported [3]. These reactions are catalyzed using catalysts based on, for example, Pd, Ti, or Co [2,4,5]. A separate group of methods for synthesizing the C=N moiety are the addition reactions of organometallic reagents to nitriles [6]. The imine moiety commonly occurs in many natural compounds, which are precursors for the synthesis of biologically active compounds, both cyclic and acyclic [7].

Today, new usages for SBs are continually being researched and developed. They are a broad class of compounds with various potential applications [8]. These compounds’ azomethine or imine groups are essential in showing excellent biological activities. SBs and their metal complexes are widely used as compounds with antimicrobial, antiviral, and anticancer properties. In the human body, the reaction of the amino group in lysine with the carbonyl groups of cofactors usually creates enzymatic intermediates with a structure characteristic of SBs [9]. Additionally, an analogous reaction occurs in rhodopsins and plays a vital role in the photoreception mechanism.

Due to the numerous applications of SBs and their various complexes, the synthesis procedure of these important ligands is still being improved. In recent years, the principles of green chemistry have increasingly been considered a good solution in SB synthesis [10]. The minimization or non-use of toxic solvents and the non-generation of residues resulting from these processes are the most important efforts of modern chemistry [11]. In the case of organic chemistry, the concept of green chemistry introduced environmentally benign protocols for the synthesis of many compounds. The use of green solvents, solvent-free synthesis, reduced energy consumption, optimized reaction yields, alternative energy sources, and the introduction of multicomponent, high efficiency, and time-saving reactions, which work at ambient temperatures, have been described [12]. The strategies using green chemistry principles in SB synthesis have been reviewed by Nagar, Raizada, and Tripathee [10].

Moreover, several methods to synthesize SBs have been described, including using natural acids as a catalyst, water as a green solvent, microwave irradiation, and eco-friendly grinding. However, most procedures of SB synthesis, even environmentally friendly ones, introduce disadvantages such as long synthesis time, high temperature, energy consumption, organic solvents, and solubility of the obtained products. Due to the low solubility in water of most of the reagents and SBs obtained, avoiding the use of harmful and toxic solvents is quite tricky. The most popular solvents, such as methanol or acetonitrile, are considered “recommended or problematic” and “problematic” due to their direct effect on humans [13]. For this reason, using water at various stages of SB synthesis, solubility, or their further transformation into compounds with new properties is becoming increasingly popular. The hydrophobic effect can lead to altered selectivity and rate accelerations. However, conducting a reaction in water may not necessarily be an improvement if several volumes of an organic solvent are needed to extract/purify the product [14].

In this study, simple, eco-friendly, and highly efficient procedure of novel Schiff bases via reaction of selected biogenic amines (BAs), such as histamine, tyramine, tryptamine, phenylethylamine, cadaverine, putrescine, spermine, and spermidine, with sodium 2-formylbenzene sulfonate and sodium 3-formylbenzene sulfonate was proposed. We used BAs because they are natural origin compounds, and their imine derivatives may have interesting properties and find various applications.

The reaction was carried out at room temperature for one hour, reducing energy consumption and saving labor, time, utilities, human health, and safety. ^1^H and ^13^C NMR, IR spectroscopy, and high-resolution mass spectrometry were used to characterize the new compounds. Additionally, the antioxidant and antibacterial activity of the obtained SBs was evaluated.

## 2. Experimental

### 2.1. Reagents and Apparatus

Analytical grade: cadaverine (Cad), putrescine (Put), spermine (Spm), spermidine (Spd), tyramine (Tyr), histamine (Him), tryptamine (Trp), 2-phenylethylamine, 99% (Phen), potassium persulfate, Mueller–Hinton agar, and methanol were purchased from Alchem (Torun, Poland). The sodium salt of 3-formyl-benzenesulfonic acid (3-FBS) was obtained by the literature procedure [15] from benzaldehyde (Sigma-Aldrich (Poznan, Poland)).

2-Formyl-benzenesulfonic acid sodium salt (2-FBS), 2,2′-azino-bis(3-ethylbenzothiazoline-6-sulfonic acid) diammonium salt (ABTS), and 6-hydroxy-2,5,7,8-tetramethylchromane-2-carboxylic acid (Trolox) were purchased from Sigma-Aldrich (Poland).

*Escherichia coli* NCTC 12923 and *Staphylococcus aureus* NCTC 12981 were purchased from Argenta S.A. (Poznan, Poland), while Cefotaxime 5, CTX5 (REF E113064), and Streptomycin 300, S300 (REF E111946) were purchased from BioMaxima S.A. (Lublin, Poland).

NMR spectra were recorded on a Bruker Avance III 400 MHz spectrometer (Bruker, Ettlingen, Germany) at 400 MHz for ^1^H and 100 MHz for ^13^C frequency resonances at 298 ± 1 K or on a Bruker Avance III 700 MHz spectrometer (Bruker, Ettlingen, Germany) at 700 MHz for ^1^H and 176 MHz for ^13^C frequency resonances at 298 ± 1 K using deuterium oxide as solvent. Chemical shifts were reported using the residual solvent peaks as references: δ 4.80 ppm for ^1^H NMR. Spin–spin coupling constants (*J*) are presented in [Hz]. Infrared spectra were recorded on a Bruker Alpha Platinum-ATR spectrometer (Bruker, Ettlingen, Germany) with OPUS 7.5 software using the ATR technique. Melting points were obtained in open-capillary tubes using electrothermal digital melting-point apparatus from Cole-Parmer Ltd. (Saint Neots, UK), and were uncorrected. UV-Vis spectra were obtained on UV-1601 spectrophotometer (Shimadzu, Kyoto, Japan). The estimation of high-resolution mass accuracy was carried out using liquid chromatography-mass spectrometry (Nexera 2 LC-3AD, Shimadzu Corp., Japan) interfaced with the time of flight as a detector (IT-TOF, Shimadzu Corp., Japan) with electrospray ionization (ESI). The mass spectrometer was operated using LCMS Solution software (LCMSsolutionVer 3, Shimadzu Corp., Japan). LC analysis was performed on a direct injection on IT-TOF by isocratic elution (60% methanol and 40% deionized water) at a constant flow rate of 0.4 mL·min^−1^.

### 2.2. Synthesis of Imines Derived from Sodium 2- or 3-Formylbenzene Sulfonate and Biogenic Amine—General Procedure

Phenylethylamine (121 mg, 1 mmol) and 2 mL of methanol were placed in a 10 mL flask. Sodium 3-formylbenzene sulfonate (208 mg, 1 mmol) was then added. The solution was stirred for 1 h at room temperature. The resulting precipitate was filtered off and dried under reduced pressure to give 290 mg of product (**1**) as a white solid, yielding 93% (mp 283–285 °C). More detailed information about the amounts of biogenic amines, sodium 2- or 3-formylbenzene sulfonate are presented in the Supplementary Materials.

### 2.3. Stability of Imines in Aqueous Solution

Phenylethylamine (12.1 mg, 0.1 mmol) and 1 mL of deuterium oxide were placed in a 2 mL flask. Sodium 2-formylbenzene sulfonate (20.8 mg, 0.1 mmol) was then added. The reaction mixture was stirred for 1 h at room temperature, and the solution was transferred to the NMR tube. ^1^H and ^13^C NMR spectra were recorded after 1 h, 2 h, 24 h, and 48 h from starting the reaction, and seven days later.

### 2.4. Antimicrobial Activity of Tested Compounds

The antimicrobial activity of the compounds obtained was determined using Gram-negative bacteria (*Escherichia coli* NCTC 12923), and Gram-positive bacteria (*Staphylococcus aureus* NCTC 12981). The bacteria strains were grown on a nutrient agar and incubated at 37 °C. All obtained SBs were dissolved in water at different concentrations: 0.01, 0.02, and 0.04 g·mL^−1^. The agar disk-diffusion method was applied for the determination of the microbial activity of the compounds tested. Mueller–Hinton agar plates were inoculated with an *Escherichia coli* (*E. coli*) and *Staphylococcus aureus* (*S. aureus*). The filter paper discs (about 5 mm in diameter), containing 5 μL of individual test compounds, were then placed on the Mueller–Hinton agar surface. The Petri dishes were then incubated for 24 h at 37 °C. Finally, the inhibition zone (mm) was measured, and the obtained results were compared with commercial antibacterial agents. The cefotaxime 5, CTX5 (REF E113064, BioMaxima S.A.), and Streptomycin 300, S300 (REF E111946 BioMaxima S.A.) antibiotic discs were used as control. Additionally, the antimicrobial activity of biogenic amines (cadaverine, putrescine, spermine, spermidine, tyramine, histamine, tryptamine, and 2-phenylethylamine), 2-FBS, and 3-FBS were tested for comparison with the titled SBs. The obtained results were presented as a mean of 15 replicates ± standard deviations (SD). One-way analysis of variation (ANOVA), followed by the Duncan test, was performed to analyze the significant differences between mean data obtained by tested biogenic amine and their derivatives (*p* < 0.05) using Statistica (Windows software package) (version 8.0, 2007).

### 2.5. ABTS Assay of Tested Compounds

The ABTS assay was performed as described by Rabiej-Kozioł [16] with some modifications and presented as a general procedure. Furthermore, detailed information about the amounts of compounds tested is collected in Table S1 (Supplementary Materials). The ABTS radical cation solution (7 mM ABTS and 2.45 mM potassium persulfate in water) was prepared and incubated in the dark for 24 h. The solution was then diluted with methanol to achieve an absorption at 734 nm of 0.700 ± 0.005. The tested polyamines and their derivatives were dissolved in methanol or water (Table S1, Supplementary Materials). Next, 0.5 mL of the obtained solutions were added to 0.1 mL of ABTS solution, made up to 10 mL with methanol or water, and remained for 10 min. The absorbance was read at 734 nm against a reagent blank. The percentage of inhibition of ABTS was calculated using the following equation.

$$\%\;\mathrm{inhibition}\;=\left[\frac{Acontrol-Asample}{Acontrol}\right]\cdot 100\%\;$$

where *A_control_* is the absorbance of control and *A_sample_* is the absorbance of the tested polyamine solution. The results obtained were presented as equivalent to the antioxidant capacity (TEAC) of Trolox (TE). For this purpose, a calibration curve was performed on the same day using six working standards of TE in methanol in the range of 2.5 × 10^−7^–5.0 × 10^−6^ mmol/mL, resulting in the determination coefficient 0.9937 (Figure S1, Supplementary Materials).

## 3. Results and Discussion

### 3.1. Structural Characteristics of the Imines Obtained

**Figure 1:**
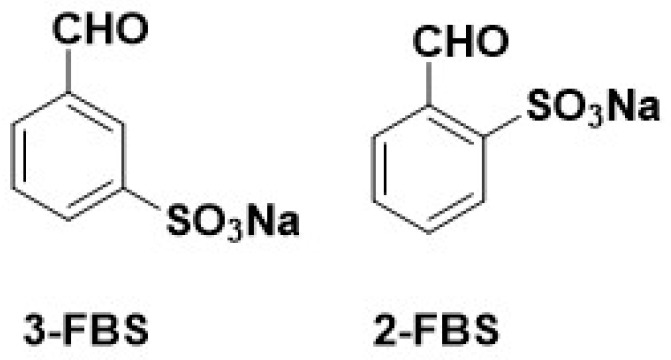
Structure of 3- and 2-FBS.

The water-soluble biogenic amine derivatives were obtained in reactions of selected BAs with sodium 2-formylbenzene sulfonate and sodium 3-formylbenzene sulfonate (Figure 1).

A stoichiometric ratio of reagents, 2-FBS and 3-FBS, was used for BAs containing one primary amine group in their structure, phenylethylamine (Phen), tryptamine (Trp), tyramine (Tyr), and histamine (Him). However, for polyamines cadaverine (Cad), putrescine (Put), spermidine (Spd), and spermine (Spm) that contain two primary amine groups in their structure, two equivalents of the sulfonates were used. The reactions were carried out in methanol at room temperature for 1 h due to the ease of isolation of the obtained products. Most of the products obtained during the reaction were in the form of insoluble precipitates in methanol, which, after the synthesis was complete, were filtered off under reduced pressure and then dried in a desiccator under vacuum.

**Figure 2:**
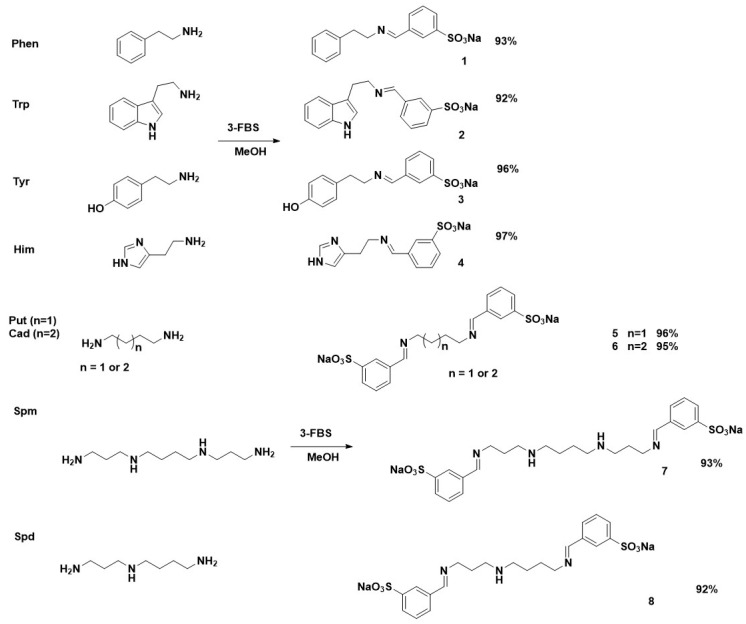
The reaction products of BAs with 3-FBS.

**Figure 3:**
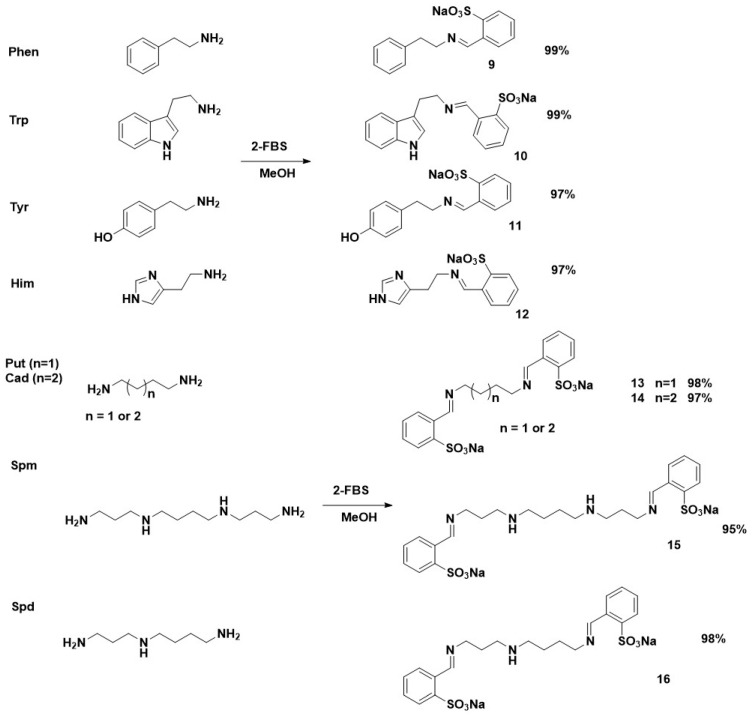
The reaction products of BAs with 2-FBS.

All BA derivatives with 2-FBS or 3-FBS were obtained in high yields (92–97% for 3-FBS products, 95–99% for 3-FBS derivatives). The structures of the obtained SBs are shown in Figure 2 and Figure 3.

The structures of all obtained SBs were confirmed by ^1^H, ^13^C NMR, HR-MS, and FTIR ATR analyses. The ^1^H and ^13^C NMR spectra clearly show characteristic signals for the imine moiety. For derivatives obtained in reaction with 3-FBS, the imine proton (–N=CH) has a chemical shift in the 7.87–8.34 ppm range. However, for products from 2-FBS, the proton signal –N=CH occurs in the range 8.81–9.02. A slightly different position for the imine carbon atom is also noticeable in the ^13^C NMR spectra, approximately 163 ppm for 3-FBS derivatives and 162 ppm for 2-FBS derivatives, respectively. The tested polyamines can be divided into symmetrical Put, Cad, and Spm, as well as asymmetrical Spd. Put, Cad, and Spm gave symmetrical, doubly substituted derivatives (**5**, **6**, **7**, **13**, **14**, **15**), visible in the NMR spectra by only one signal of a proton and an imine carbon. However, the spectra of Spd derivatives (**8** and **16**) clearly show two imine groups (e.g., for **8**: 8.00 and 8.15 ppm).

A full description of the obtained spectra for all products is presented in the Supplementary Materials.

### 3.2. Stability in Water

The reaction of a primary amine with an aldehyde is associated with the release of a water molecule; so, very often, during the synthesis of imines, it is removed by azeotropic distillation or by adding a water-capturing agent, such as molecular sieves or anhydrous magnesium sulphate. Imines are typically unstable in aqueous solutions and hydrolyzed by acids, bases, and metal ions [17]. However, macrocycle imines are also known to be stable in an aqueous environment [18]. Therefore, we decided to investigate the synthesis and stability of imine derivatives of sodium formylbenzene sulfonates in water.

**Figure 4:**
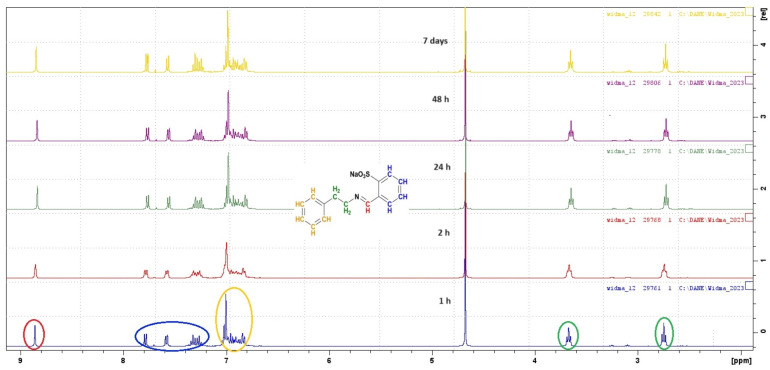
^1^H NMR of compound **9** after 1 h (blue spectra), 2 h (red spectra), 24 h (green spectra), 48 h (purple spectra), and 7 days (yellow spectra) from starting the reaction. The circles on the spectrum correspond to the colors of hydrogen atoms in the presented structure.

**Figure 5:**
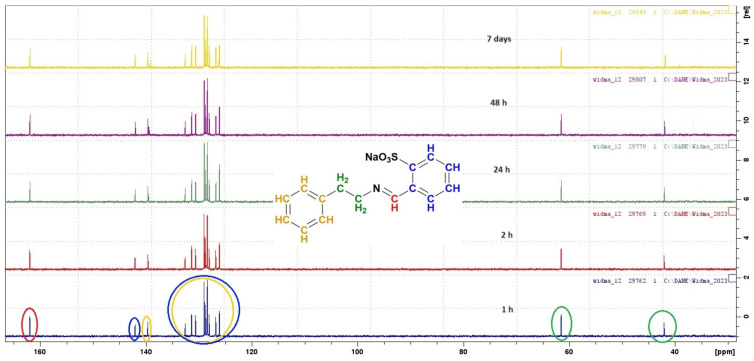
^13^C NMR of compound **9** after 1 h (blue spectra), 2 h (red spectra), 24 h (green spectra), 48 h (purple spectra), and 7 days (yellow spectra) from the start of reaction. The circles on the spectrum correspond to the colors of carbon atoms in the presented structure.

To verify the stability in water of the obtained SBs, we performed the synthesis of the Phen derivative in reaction with 2-FBS in deuterated water. The resulting mixture was transferred to an NMR tube to record ^1^H and ^13^C NMR spectra (Figure 4 and Figure 5).

The first spectrum was recorded one hour after the start of the reaction. In the ^1^H NMR spectrum, a characteristic signal from the proton in the carbon atom of the imine group (CH=N) can be observed at approximately δ 8.80 ppm. Moreover, the spectrum includes signals coming from alkyl protons in the Phen part of the structure and signals coming from hydrogen atoms from aromatic rings in the phenylethylamine part and to the benzenesulfonate system in the δ range of 6.80–7.80 ppm. The spectra were recorded consecutively after 2 h, 24 h, 48 h, and 7 days. Between the spectra recording, the sample in the NMR tube was stored at 4 °C. ^13^C spectra were prepared similarly. Each of the registered spectra shows a signal coming from the carbon atom in the imine group (HC=N) with a chemical shift of approximately δ 160 ppm (Figure 5). Based on the recorded spectra, it can be concluded that the synthesis of new SBs can be carried out in aqueous solutions and that they are stable in aqueous solutions up to 7 days.

### 3.3. Antimicrobial Activity

The exceptional popularity of SBs can be attributed to their wide range of biological activities, including antiviral, antifungal, and antibacterial properties. The imine or azomethine group (>C=N–) seems critical for their biological activities. On the other hand, in most cases, higher activity has been reported for SB–metal complexes rather than SBs alone [19]. Moreover, aryl or alkyl substituents at the imine bond may affect the final antibacterial activity of SBs. In this study, we proposed selecting BAs as reagents for synthesizing new SBs. It should be noted that the antibacterial properties of BAs are discussed in the literature [20,21,22]. The SBs obtained and the reagents (BAs, 2-FBS, and 3-FBS) were individually tested as an antibacterial agent against *E. coli* and *S. aureus*. The *S. aureus* is a Gram-positive round-shaped bacterium and is a usual member of the body microbiota, while *E. coli* is a Gram-negative rod-shaped bacterium in either skin, such as traumatic wounds, decubitus, and foot ulcers [23]. For this purpose, we applied a simple, fast, and commonly used agar-disk diffusion method to estimate antimicrobial activity. After an incubation period, the extent of the inhibition area around the disk was quantified. Unfortunately, of all the tested SBs, only derivatives of Phen (compounds **1**, **9**), Spd (compounds **8**, **16**), and Spm (compounds **7**, **15**) were characterized by microbiological activity.

Furthermore, inhibition of bacterial growth (both *E. coli* and *S. aureus*) was only observed at a concentration of 0.04 g·mL^−1^ (200 μg per disk) (Figure S2, Supplementary Materials). Both 2-FBS and 3-FBS did not inhibit the growth of tested bacteria. However, among the tested BAs, only Phen, Spm, and Spd showed a significant area of inhibition of bacterial growth around the disks in the agar medium. The results obtained are listed in Table 1, while Figure S3 (Supplementary Materials) illustrates the inhibition zone around the disc loaded with the selected, tested samples against the two bacteria.

The Spm solution has a zone of inhibition diameter of 22.10 mm and 23.62 mm against *E. coli* and *S. aureus*, respectively. In case of Spd solution, an area of inhibition of 20.76 mm in diameter is observed against *S. aureus*, while *E. coli* was shown to be resistant. It should be added that the values of inhibition of the bacterial growth zone around the disc with an aqueous Spm and Spd solutions (200 µg) were comparable to those obtained for cefotaxime (5 µg) and streptomycin (300 µg).

The results obtained for the antimicrobial activity of Spd, Spm, cefotaxime, and streptomycin were compared by one-way ANOVA followed by Duncan’s test (*p* < 0.05). The mean value of the zone inhibition growth of *E. coli* and *S. aureus* for Spm did not show significant differences compared to the antibiotics tested. In contrast, the antimicrobial activity of Spd differed significantly from that of inhibiting the growth of *E. coli* by tested antibiotics. Additionally, both discussed polyamines are present in all organism cells, where they play a fundamental role in cell proliferation and have both pro- and anti-apoptotic effects. Transport systems efficiently absorb exogenous polyamines, and as endogenous ones, they can also affect different biological targets.

The uniform distribution of positive charges across a hydrophobic backbone is essential in how these compounds exert their diverse functions. Moreover, the multiple activities induced by polyamines due to their interaction with different biological targets support the proposal of the polyamine skeleton as a universal model for further modification. According to Inclán et al. [20], polyamine research has become an essential field for drug development. Their potential application has been studied as anticancer and antiproliferative agents, agonist–antagonist receptor ligands, or antiparasitic compounds. The positive charges of the amino groups have been considered a critical factor in their antimicrobial activity. The latter was probably the reason for the observed activity of both polyamines in inhibiting the growth of *E. coli* and *S. aureus*. However, *S. aureus* is a peculiar example, as it lacks polyamine biosynthetic genes and displays hypersensitivity to exogenously added polyamines [21].

The obtained compounds (**7**, **8**, **15**, **16**, Table 1) were characterized by significantly lower values (Duncan test) of the growth inhibition zone for both examined bacterial strains compared to polyamines. The decrease in the antibacterial activity of new SBs may have resulted from blocking amino groups in polyamines. Nevertheless, observed mean values for the diameter of the inhibition zone (mm) were statistically significantly similar for both derivatives of Spm or Spd with 2-FBS and 3-FBS (Duncan test, Table 1).

The antibacterial activity of compounds **7** and **15** was more pronounced against Gram-positive bacteria (*S. aureus)* than Gram-negative bacteria (*E. coli*), which could be due to their cell wall constituents and structure. According to [8], the outer membrane of Gram-negative bacteria serves as a permeability barrier that controls the entry and exit of various substances and contributes to osmoprotection. Although Gram-positive bacteria possess a thicker peptidoglycan layer, they lack a protective outer membrane. Therefore, Gram-positive bacteria are more susceptible to antimicrobial agents than Gram-negative bacteria.

Phen was another amine that showed an inhibitory effect on both *S. aureus* and *E. coli*. Yet, the measured growth inhibition values around the discs were much lower and were as follows: 11.47 mm and 12.32 mm. This amine has been discussed to have antimicrobial activity against bacteria such as *Escherichia coli*, *Pseudomonas aeruginosa*, and *Staphylococcus aureus* in settings such as beef, biofilms, and clinical environments [10]. Moreover, this simplest aromatic amine provides the basic chemical structure for several classes of drugs with broad biological activity. For this reason, its derivatives have attracted intense interest. For many years, they have emerged as a promising biologically active scaffold in the search for new agents against many diseases [24]. Our results indicated that compounds **1** and **9** displayed statistically significantly similar (Duncan test, Table 1) antimicrobial activity against *S. aureus* and *E. coli* as Phen.

In general, our results demonstrated that adding imine–phenylsulfonate groups to the polyamine backbone decreased the efficacy of the resulting compounds. The latter suggested that the total charge and polyamine topology determined their antimicrobial activity. However, this effect was not observed for Phen, which is particularly important in case of *S. aureus* infections. Because of the lack of an outer membrane, Gram-positive bacteria are highly subject to antibacterial activities. However, recently, there has been discussion about the emergence of antibiotic-resistant strains such as methicillin-resistant *S. aureus* [25]. Therefore, searching for new compounds with potential antibacterial properties and low toxicity remains relevant.

The inhibition of bacterial growth observed in this study by selected biogenic amine derivatives is not synonymous with their death. Moreover, the applied disk-diffusion assay cannot distinguish bactericidal and bacteriostatic effects. Therefore, further studies on the potential application of the new compounds obtained as antibacterial agents should be continued.

### 3.4. Antioxidant Activity (AA)

**Figure 6:**
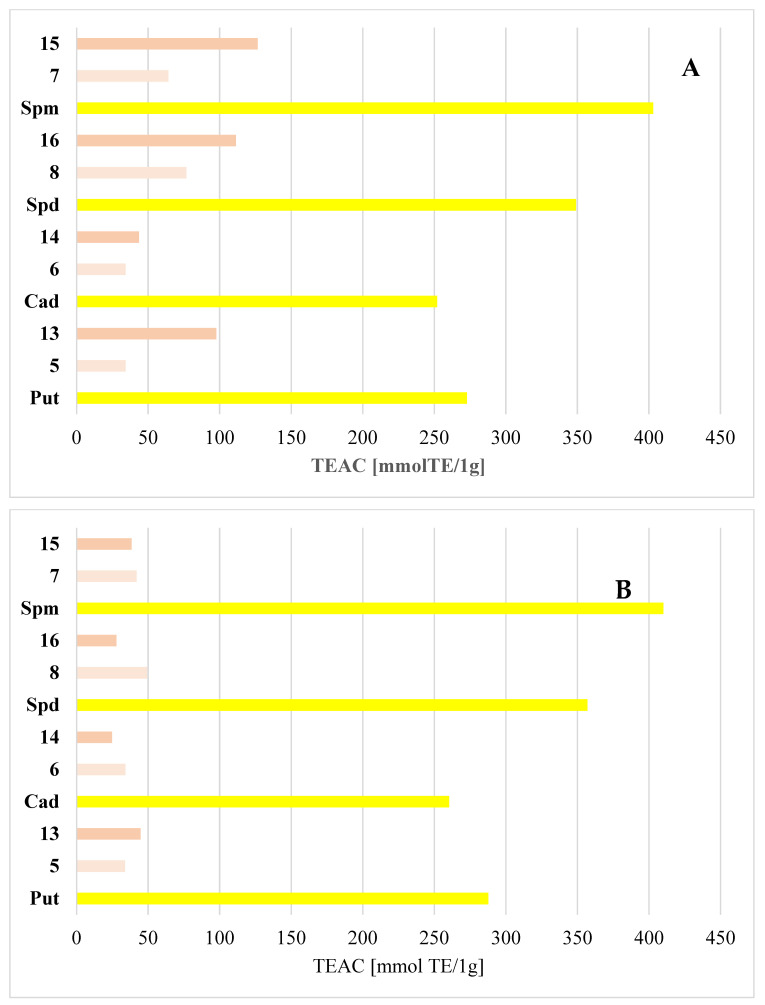
Antioxidant activity of polyamines and their derivatives with 2-FBS and 3-FBS, where (**A**) reactions carried out in methanol; (**B**) reactions carried out in water.

Nowadays, oxidative stress is well known to play a critical role in premature aging, contributing to progressive loss of tissue and organ function. This phenomenon arises from an imbalance between oxidative and reductive processes during physiological metabolism [26]. Because most Schiff bases contain hydroxyl groups in their chemical structure, they can block the harmful effects of free radicals from antioxidants. For this reason, the following research stage was to evaluate the antioxidant activity (AA) of the derivatives obtained using the ABTS test. The ABTS assay determines the AA scavenging capacity by reacting with a potent antioxidant agent (potassium persulfate) in the presence of ABTS salt. This assay is a simple and frequent approach based on inhibiting ABTS formation by one-electron oxidants. AA research was carried out for selected SBs obtained by the reaction of polyamines (Put, Cad, Spm, and Spd) with 2-FBS and 3-FBS. The results obtained are presented in Figure 6.

Considering the obtained results, it is evident that all polyamines tested, both in water and in methanol, showed antioxidant activity, consistent with the information presented in the literature [27,28,29]. Natural polyamines, such as Put, Spd, and Spm, are present in all organism cells, where they play a fundamental role in cell proliferation and have both pro- and anti-apoptotic effects. According to Vrijsen et al. [29], polyamines are protected under oxidative stress conditions. They stimulate the expression of different factors in the antioxidative response, such as catalase and glutathione, but they also function as antioxidants. These compounds could act as antioxidants, as their anion- and cation-binding properties involve radical scavenging. They have been shown to inhibit lipid peroxidation and metal-catalyzed induction of oxidative stress.

Moreover, polyamines are positively charged at a physiological pH and thus behave as polycationic molecules and exhibit increased hydrophilicity and flexibility with an increasing number of charged amino groups. Following this idea, the more amino groups, the higher the antioxidant capacity. Similar results were obtained in this study. The antioxidant capacity of tested polyamines decreased with the number of primary and secondary amino groups. The best results were obtained for Spm: 403 μmol TE/1 g and 410 μmol TE/1 g in methanol and water solution, respectively, while Cad was characterized by the lowest TEAC values (252 and 260 μmol TE/1 g, respectively). Spm functions as a free radical scavenger that is oxidized during this process, thus forming spermine dialdehyde. Its role in protecting cells from the action of free radicals has been discussed and studied for many years [30]. For example, the potential of polyamines to control free radicals was investigated and Spm was found, compared to Spd, to have a more remarkable ability to control H_2_O_2_ in cultured cells. However, the roles of polyamines in the elimination of free radicals remain unclear.

The radical scavenging capacity of polyamine SBs was lower under the tested conditions than parent compounds. Regardless of the reagents, the antioxidant activity of the obtained SBs increased with the number of amino groups, just as in the case of polyamines. The latter suggests that the lower TEAC values for the SBs may have resulted from blocking primary amino groups at the ends of the aliphatic chain. The probable step of terminating reactive radicals by polyamines is hydrogen atom transfer from the antioxidant molecule to the reactive radical. The relatively low bond dissociation enthalpy of the N-H bond in amines enhances the antioxidant potency. Differences in the antioxidant capacity values for the obtained SBs could also result from the position of the sulfonyl group relative to nitrogen. In case of the meta position, significantly higher TEAC values were observed for methanolic solutions. A similar situation was not observed for aqueous solutions, where the ortho position of the sulfonyl group improved antioxidant properties.

The most essential quality of polyamines is that they play a significant role in the normal functioning of tissues with high rates of cell renewal and carcinogenesis. Ongoing intensive research on their complexes with various compounds has shown that polyamine derivatives may find applications as therapeutic drugs with a broad spectrum of pharmacological activity [31]. Therefore, further research on these compounds can expand the understanding of the possibilities of SBs ligands and their applications in analytical chemistry, heterogenous and homogeneous catalysis, or medicine.

## 4. Future Prospects and Conclusions

We note that the synthesis of new SBs ligands is still attractive because it exhibits comprehensive analytical, biological, physico-chemical, catalytical, and structural properties [32,33,34]. For example, easily accessible polydentate SBs and their metal complexes are essential in pharmacy and medicine [35]. Developing novel SBs as ligands can effectively fight against the current antimicrobial, anticancer, and antioxidant resistance threat.

This paper proposes a simple, eco-friendly, and high-efficiency procedure for synthesizing new SBs based on biogenic amines and sodium 2-formylbenzene sulfonate and 3-formylbenzene sulfonate. The proposed procedure meets the requirements of green synthesis because it significantly reduces energy consumption. The obtained compounds are soluble in water, substantially reducing the consumption of organic solvents. Notably, the obtained SBs are stable in aqueous solutions for at least seven days, allowing their usage in analytical procedures. Due to their physicochemical properties (solubility and stability in water, change in polarity, structural properties), the obtained compounds can be used for chromatographic analysis of biogenic amines in complex matrices, such as food samples. Moreover, based on their antimicrobial and antioxidative activity and ability to form complexes with various metal ions, they can be considered promising for future applications in biology, catalysis, or designing newer therapeutic agents [35,36].

## Figures and Tables

**Table 1:** The mean values of inhibition zone diameter [mm] ± standard deviations (SD) for BAs, 2-FBS, 3-FBS, and SBs.

	Phen	1	9	Spd	8	16	Spm	7	15	CFT	SPT
*S. aureus*	11.47 ^A^ ±0.37	11.42 ^A^ ±0.58	11.29 ^A^ ±0.58	20.79 ^B^ ±0.58	16.51 ^A^ ±0.92	16.05 ^A^ ±0.65	23.62 ^B^ ±1.63	17.56 ^A^ ±1.81	16.91 ^A^ ±1.48	22.96 ±0.63	22.27 ±0.32
*E. coli*	12.32 ^A^ ±0.42	12.25 ^A^ ±0.36	12.08 ^A^ ±0.65	-	-	-	22.10 ^B^ ±2.18	9.22 ^A^ ±0.64	8.76 ^A^ ±0.52	21.86 ±0.40	22.15 ±1.10

CFT—Cefotaxime; SPT—Streptomycin; the concentration of the tested compounds was 0.04 g·mL^−1^ (200 μg per disc); different letters (A,B) within the same row for amine and its derivatives indicate significant differences (one-way ANOVA and Duncan test, *p* < 0.05); sorted from the lowest to the highest values, where “A” was the lowest.

## Data Availability

Data are contained within the article.

## Supplementary Materials

The following supporting information can be downloaded at: https://www.mdpi.com/article/10.3390/ma17020520/s1, Physicochemical data for all obtained SBs, including yields, melting points, HR-MS, NMR, and IR spectra description are presented. Table S1. The masses and concentrations of polyamines and derivatives obtained used in the ABTS procedure; Figure S1. The calibration curve for ABTS assay; Figure S2. The inhibition zones of compounds 7 for *E. coli*; where (A) concentration 0.01 g·mL^−1^; (B) concentration 0.02 g·mL^−1^; (C) concentration 0.04 g·mL^−1^; Figure S3. The inhibition zones of compounds: 9 for *E. coli* (A); 1 for *S. aureus* (B); 9 for *S. aureus* (C), Phen for *E. coli* (D) and *S. aureus* (E); ^1^H and ^13^C NMR, ESI-MS spectras.
