# Supplementary material for: New Water-Soluble (Iminomethyl)benzenesulfonates Derived from Biogenic Amines for Potential Biological Applications
Source: Materials (Basel). 2024 Jan 22;17(2):520. doi: 10.3390/ma17020520 (PMC10817586; doi:10.3390/ma17020520)

New Water-Soluble (Iminomethyl)benzenesulfonates Derived from Biogenic Amines for Potential Biological Applications

Anna Kmieciak [1,*], Marek P. Krzemiński [1], Anastasiia Hodii [1], Damian Gorczyca [2,3] and Aneta Jastrzębska [1,*]

[1] Faculty of Chemistry, Nicolaus Copernicus University in Torun, 7 Gagarin Str., 87-100 Torun, Poland; mkrzem@umk.pl (M.P.K.); anastasiagodiy@gmail.com (A.H.)
[2] Faculty of Medicine, Lazarski University, 43 Świeradowska Str., 02-662 Warsaw, Poland; damian.gorczyca@lazarski.pl
[3] LymeLab Pharma, Kochanowskiego 49A Str., 01-864 Warsaw, Poland
* Correspondence: akmieciak@umk.pl (A.K.); aj@umk.pl (A.J.)

SUPPORTING INFORMATION

Table S1. The masses and concentrations of polyamines and obtained derivatives used in the ABTS procedure.

	**Put**	**Cad**	**Spd**	**Spm**	**5**	**6**	**7**	**8**	**13**	**14**	**15**	**16**
Mass [mg]	11.8	10.4	12.4	10.3	10.6	10.4	10.3	11.0	11.0	10.4	10.9	11.7
C [mol/L]	$0.1339 \cdot 10^{-1}$	$0.1018 \cdot 10^{-1}$	$0.8537 \cdot 10^{-2}$	$0.5090 \cdot 10^{-2}$	$0.1568 \cdot 10^{-2}$	$0.1507 \cdot 10^{-2}$	$0.1770 \cdot 10^{-2}$	$0.2095 \cdot 10^{-2}$	$0.2350 \cdot 10^{-2}$	$0.2157 \cdot 10^{-2}$	$0.2150 \cdot 10^{-2}$	$0.2074 \cdot 10^{-2}$

Synthesis of imines derived from sodium 2- or 3-formylbenzene sulfonate and biogenic amine - general procedure

Sodium (*E*)-3-((phenethylimino)methyl)benzenesulfonate (1)

Phenylethylamine (121 mg, 1 mmol) and 2 mL of methanol were placed in a 10 mL flask. Sodium 3-formylbenzenesulfonate (208 mg, 1 mmol) was then added. The reaction mixture was stirred for 1 hour at room temperature. The resulting precipitate was filtered off and dried under reduced pressure. 290 mg of product was obtained with a yield of 93% in the form of a white solid (mp 283-285 °C).

^{1}H NMR (700 MHz, D_2O) δ ppm 2.97 (t, *J*=6.78 Hz, 2 H), 3.83 (td, *J*=6.78, 1.08 Hz, 2 H), 7.17 - 7.29 (m, 5 H), 7.53 (m, 1 H), 7.71 (m, 1 H), 7.81 - 7.85 (m, 1 H), 7.93 (m, 1 H), 8.03 (s, 1 H).

^{13}C NMR (176 MHz, D_2O) δ ppm 36.04, 61.35, 126.35, 127.88, 128.55, 128.73, 129.31, 129.58, 130.56,135.74, 139.70, 143.16, 163.26.

FTIR (ATR) (cm^{-1}): 3061.45, 3026.92, 2932.30, 2851.81, 2834.85, 1648.61, 1575.91, 1497.40, 1475.81, 1456.44, 1422.24, 1361.78, 1342.01, 1280.12, 1261.71, 1233.55, 1186.52, 1125.15, 1084.44, 1050.75, 1013.10, 994.88, 959.14, 930.64, 903.93, 886.72, 812.60, 753.06, 735.73, 698.86, 688.28, 652.13, 627.60, 603.87, 585.52, 566.65, 528.70, 501.18, 466.57, 430.70, 421.04.

HRMS (ESI-TOF) *m/z*: $[M + H]^+$ Calcd for: $C_{15}H_{14}NNaO_3S$ Molecular Weight: 312.0670; found 312.0565.

Sodium (*E*)-3-(((2-(1*H*-indol-3-yl)ethyl)imino)methyl)benzenesulfonate (2)

Tryptamine (160 mg, 1 mmol) and sodium 3-formylbenzenesulfonate (208 mg, 1 mmol) were taken to reaction. Product was obtained with 92% yield (322 mg, mp 261-263 °C).

^{1}H NMR (700 MHz, D_2O) δ ppm 3.11 (t, *J*=6.56 Hz, 2 H), 3.85 (t, *J*=6.45 Hz, 2 H), 7.04 - 7.08 (m, 1 H), 7.09 (m, 1 H), 7.12 - 7.15 (m, 1 H), 7.40 (m, 1 H), 7.48 (m, 1 H), 7.58 - 7.61 (m, 1 H), 7.63 (m, 1 H), 7.77 - 7.82 (m, 1 H), 7.83 (m, 1 H), 7.86 (s, 1 H), 8.06 (br.s., 1 H).

^{13}C NMR (176 MHz, D_2O) δ ppm 25.42, 60.68, 111.67, 112.28, 118.87, 119.00, 121.68, 123.72, 126.98, 127.69, 129.41, 129.99, 130.63, 135.69, 136.14, 143.04, 162.75.

HRMS (ESI-TOF) *m/z*: [M - Na]$^-$ Calcd for: $C_{17}H_{15}N_2NaO_3S$ Molecular Weight: 327.0803; found 327.0758.

FTIR (ATR) (cm^{-1}): 3426.53, 3073.01, 3055.29, 2924.63, 2851.17, 2831.04, 2114.42, 2021.78, 1645.55, 1575.55, 1475.26, 1454.5, 1420.03, 1356.98, 1334.19, 1279.76, 1233.76, 1186.17, 1124.80, 1085.42, 1051.27, 1036.57, 1011.88, 994.89, 957.92, 931.53, 903.83, 887.80, 871.48, 815.28, 767.26, 734.08, 687.93, 655.87, 626.66, 602.91, 584.63, 574.70, 566.01, 526.90, 475.65, 424.25, 409.11.

Sodium (*E*)-3-(((4-methylphenethyl)imino)methyl)benzenesulfonate (3)

Tyramine (137 mg, 1 mmol) and sodium 3-formylbenzenesulfonate (208 mg, 1 mmol) were taken to reaction. Product was obtained with 96% yield (314 mg, mp 217-219 °C).

^{1}H NMR (700 MHz, D_2O) δ ppm 2.73 (t, *J*=6.88 Hz, 2 H), 3.62 (t, *J*=6.88 Hz, 2 H), 6.64 (m, 2 H), 6.95 (m, 2 H), 7.43 (m, 1 H), 7.56 (m, 1 H), 7.77 (m, 1 H), 7.84 (m, 1 H), 7.87 (s, 1 H).

^{13}C NMR (176 MHz, D_2O) δ ppm 41.32, 61.70, 116.19, 124.83, 129.57, 130.17, 130.47, 130.65, 135.77, 143.21, 156.63, 159.10, 163.07.

HRMS (ESI-TOF) *m/z*: [M - Na]$^-$ Calcd for: $C_{15}H_{14}NNaO_4S$ Molecular Weight: 304.0644; found 304.0610.

FTIR (ATR) (cm^{-1}): 3013.30, 2927.14, 2112.15, 1644.01, 1562.41, 1513.48, 1413.22, 1341.25, 1190.42, 1116.19, 1041.28, 995.82, 963.12, 926.34, 825.81, 799.14, 726.52, 688.25, 651.00, 616.81, 552.02, 511.32, 465.44.

Sodium (*E*)-3-(((2-(1H-imidazol-4-yl)ethyl)imino)methyl)benzenesulfonate (4)

Histamine (111 mg, 1 mmol) and sodium 3-formylbenzenesulfonate (208 mg, 1 mmol) were taken to reaction. Product was obtained with 97% yield (292 mg, mp 166-168 °C).

^{1}H NMR (400 MHz, D_2O) δ ppm 2.71 - 2.78 (m, 2 H), 3.74 (t, *J*=6.60 Hz, 2 H), 7.41 (m, 1 H), 7.44 - 7.50 (m, 1 H), 7.55 (m, 1 H), 7.66 (m, 1 H), 7.78 (m, 1 H), 7.90 (s, 1 H), 8.02 (s, 1 H).

^{13}C NMR (101 MHz, D_2O) δ ppm 40.20, 59.47, 117.16, 124.88, 130.62, 131.50, 135.10, 135.74, 135.88, 142.40, 143.16, 163.47.

HRMS (ESI-TOF) *m/z*: $[M + H]^+$ Calcd for: $C_{12}H_{12}N_3NaO_3S$ Molecular Weight: 302.0575; found 302.0475.

FTIR (ATR) (cm^{-1}): 3118.51, 2928.18, 2853.91, 1643.35, 1566.27, 1513.93, 1489.49, 1413.48, 1340.45, 1186.61, 1114.87, 1037.35, 996.03, 924.14, 799.64, 725.48, 690.54, 648.19, 614.75, 557.75, 519.63, 414.25.

Sodium 3,3'-((1*E*,1'*E*)-(butane-1,4-diylbis(azanylylidene))bis(methanylylidene))dibenzene-sulfonate (5)

Putrescine (88 mg, 1 mmol) and sodium 3-formylbenzenesulfonate (416 mg, 2 mmol) were taken to reaction. Product was obtained with 96% yield (450 mg, mp >300 °C).

^{1}H NMR (400 MHz, D_2O) δ ppm 1.61 - 1.72 (m, 4 H), 3.51 - 3.66 (m, 4 H), 7.50 - 7.66 (m, 2 H), 7.75 - 7.83 (m, 2 H), 7.84 - 7.92 (m, 2 H), 7.98 - 8.12 (m, 2H), 8.34 (s, 2 H).

^{13}C NMR (101 MHz, D_2O) δ ppm 27.37, 59.84, 124.96, 127.89, 129.67, 130.67, 135.87, 143.18, 162.94.

HRMS (ESI-TOF) *m/z*: $[M - Na]^-$ Calcd for: $C_{18}H_{18}N_2Na_2O_6S_2$ Molecular Weight: 445.0504; found 445.0511.

FTIR (ATR) (cm^{-1}): 3596.66, 3520.98, 3053.25, 2940.40, 2847.02, 2323.98, 2191.05, 2168.23, 2129.81, 2056.80, 2002.70, 1958.76, 1648.96, 1619.36, 1568.73, 1416.08, 1344.75, 1288.06, 1208.61, 1171.16, 1123.62, 1082.89, 1041.71, 994.71, 965.98, 922.07, 884.60, 800.70, 744.63, 687.81, 652.52, 624.51, 566.03, 517.11, 463.85, 452.40, 427.85.

Sodium 3,3'-((1*E*,1'*E*)-(pentane-1,5-diylbis(azanylylidene))bis(methanylylidene))dibenzene-sulfonate (6)

Cadaverine (102 mg, 1 mmol) and sodium 3-formylbenzenesulfonate (416 mg, 2 mmol) were taken to reaction. Product was obtained with 95% yield (458 mg, mp 282-284 °C).

^{1}H NMR (700 MHz, D_2O) δ ppm 1.31 - 1.42 (m, 2 H), 1.56 (td, *J*=15.00, 7.42 Hz, 4 H), 3.47 (t, *J*=6.78 Hz, 4 H), 7.41 (m, 1 H), 7.49 - 7.55 (m, 1 H), 7.58 (m, 1 H), 7.72 (m, 1 H), 7.77 (m, 1 H), 7.82 (m, 1 H), 7.94 (s, 1 H), 7.99 (s, 1 H), 8.17 (s, 1 H), 8.23 (s, 1 H).

^{13}C NMR (176 MHz, D_2O) δ ppm 23.69, 29.47, 60.14, 124.82, 124.92, 127.91, 127.93, 129.69, 129.71, 130.58, 130.73, 135.76, 135.92, 143.30, 143.35, 162.64, 162.67.

HRMS (ESI-TOF) *m/z*: $[M - Na]^-$ Calcd for: $C_{19}H_{20}N_2Na_2O_6S_2$ Molecular Weight: 459.0660; found 459.0669.

FTIR (ATR) (cm^{-1}): 3363.31, 2929.71, 2852.04, 1646.52, 1569.77, 1414.99, 1347.39, 1332.14, 1216.90, 1186.82, 1123.87, 1081.24, 1038.22, 1012.60, 995.18, 955.41, 922.98, 824.31, 802.35, 776.64, 749.11, 731.45, 689.43, 648.34, 617.65, 565.45, 532.51, 513.74, 461.90, 436.50.

Sodium 3,3'-((1*E*,15*E*)-2,6,11,15-tetraazahexadeca-1,15-diene-1,16-diyl)dibenzenesulfonate (7)

Spermine (202 mg, 1 mmol) and sodium 3-formylbenzenesulfonate (416 mg, 2 mmol) were taken to reaction. Product was obtained with 93% yield (542 mg, mp 199-201 °C).

^{1}H NMR (700 MHz, D_2O) δ ppm 1.60 - 1.75 (m, 2 H), 1.90 - 2.01 (m, 4 H), 2.97 (m, 8 H), 3.97 (m, 4 H), 7.78-7.79 (m, 1H), 7.83 - 7.87 (m, 1 H), 7.96 (m, 2 H), 8.04 (m, 4 H), 8.18 (s, 1 H), 8.25 (s, 1 H).

^{13}C NMR (176 MHz, D_2O) δ ppm 23.42, 25.12, 44.28, 49.00, 69.13, 127.89, 129.11, 129.67, 131.74, 140.98, 143.12, 162.11.

HRMS (ESI-TOF) *m/z*: $[M + H]^+$ Calcd for: $C_{24}H_{32}N_4Na_2O_6S_2$ Molecular Weight: 583.1637; found 583.1470.

FTIR (ATR) (cm^{-1}): 3401.56, 3275.80, 2937.08, 2851.28, 2112.50, 1638.02, 1561.95, 1409.32, 1182.93, 1113.60, 1033.97, 995.87, 923.76, 799.59, 725.07, 691.72, 645.27, 614.74, 512.62.

Sodium 3-((*E*)-((3-((4-(((E)-3-sulfonatobenzylidene)amino)butyl)amino)propyl)imino)methyl) benzenesulfonate (8)

Spermidine (145 mg, 1 mmol) and sodium 3-formylbenzenesulfonate (416 mg, 2 mmol) were taken to reaction. Product was obtained with 92% yield (484 mg, mp 268-270 °C).

^{1}H NMR (400 MHz, D_2O) δ ppm 1.19 - 1.30 (m, 2 H), 1.66 (m, 2 H), 1.94 - 2.05 (m, 2 H), 2.18 - 2.29 (m, 2 H), 2.70 (m, 2 H), 2.99 - 3.11 (m, 2 H), 3.11 - 3.26 (m, 2 H), 7.52 - 7.62 (m, 3 H), 7.73 - 7.83 (m, 4 H), 7.90 (m, 1 H), 8.00 (s, 1 H), 8.15 (s, 1 H).

^{13}C NMR (101 MHz, D_2O) δ ppm 23.37, 25.14, 29.50, 48.95, 51.72, 52.97, 59.64, 124.44, 124.93, 129.08, 129.55, 129.66, 129.72, 130.43, 130.87, 141.04, 141.12, 143.05, 143.29, 162.65, 162.69.

HRMS (ESI-TOF) *m/z*: $[M + H]^+$ Calcd for: $C_{21}H_{26}N_3Na_2O_6S_2$ Molecular Weight: 526.1058; found 526.1060.

FTIR (ATR) (cm^{-1}): 3432.84, 2934.25, 2856.12, 2323.79, 2129.55, 1643.64, 1568.95, 1416.63, 1188.09, 1117.03, 1038.52, 1013.62, 996.52, 923.03, 797.40, 728.15, 692.18, 647.64, 615.07, 559.08, 525.78, 463.25.

Sodium (*E*)-2-((phenethylimino)methyl)benzenesulfonate (9)

Phenylethylamine (121 mg, 1 mmol) and sodium 2-formylbenzenesulfonate (208 mg, 1 mmol) were taken to reaction. Product was obtained with 99% yield (308 mg, mp 90-92 °C).

^{1}H NMR (400 MHz, D_2O) δ ppm 2.82 (t, *J*=7.04 Hz, 2 H), 3.75 (t, *J*=6.75 Hz, 2 H), 7.01 - 7.13 (m, 5 H), 7.30 - 7.45 (m, 2 H), 7.62 - 7.70 (m, 1 H), 7.86 (m, 1 H), 8.93 (s, 1 H).

^{13}C NMR (101 MHz, D_2O) δ ppm 42.07, 61.52, 126.13, 126.80, 128.40, 129.01, 130.62, 131.37, 132.60, 139.61, 139.65, 142.06, 161.95.

HRMS (ESI-TOF) *m/z*: $[M + H]^+$ Calcd for: $C_{15}H_{14}NNaO_3S$ Molecular Weight: 312.0670; found 312.0571.

FTIR (ATR) (cm^{-1}): 3438.03, 3061.78, 3026.66, 2924.60, 2848.15, 2225.91, 2112.87, 1687.92, 1635.79, 1603.11, 1589.25, 1567.73, 1495.60, 1466.81, 1453.72, 1439.91, 1380.28, 1345.59, 1184.32, 1135.80, 1089.02, 1020.72, 965.98, 909.08, 845.60, 827.56, 760.76, 724.06, 697.63, 643.92, 613.25, 563.26, 499.79.

Sodium (*E*)-2-(((2-(1H-indol-3-yl)ethyl)imino)methyl)benzenesulfonate (10)

Tryptamine (160 mg, 1 mmol) and sodium 2-formylbenzenesulfonate (208 mg, 1 mmol) were taken to reaction. Product was obtained with 99% yield (347 mg, mp 133-135 °C).

^{1}H NMR (700 MHz, D_2O) δ ppm 3.13 (t, *J*=6.88 Hz, 2 H), 3.89 - 3.94 (m, 2 H), 7.11 (m, 1 H), 7.15 - 7.19 (m, 2 H), 7.41 (1 H), 7.49 - 7.55 (m, 2 H), 7.67 - 7.71 (m, 2 H), 7.81 - 7.85 (m, 1 H), 8.83 (s, 1 H).

^{13}C NMR (176 MHz, D_2O) δ ppm 25.55, 60.87, 111.69, 112.46, 118.77, 118.97, 121.69, 123.47, 126.71, 127.06, 128.14, 130.65, 131.60, 132.47, 136.16, 141.60, 161.85.

HRMS (ESI-TOF) *m/z*: $[M - Na]^-$ Calcd for: $C_{17}H_{15}N_2NaO_3S$ Molecular Weight: 327.0803; found 327.0759.

FTIR (ATR) (cm^{-1}): 3425.52, 3073.19, 3055.66, 2923.83, 2851.19, 2831.52, 2220.06, 2091.47, 1982.51, 1949.23, 1922.51, 1645.40, 1576.12, 1549.22, 1475.23, 1454.78, 1420.31, 1357.21, 1334.51, 1280.01, 1233.69, 1185.86, 1124.88, 1085.78, 1050.67, 1036.96, 994.81, 957.93, 931.42, 903.84, 887.89, 870.60, 814.92, 767.18, 734.19, 687.82, 655.50, 619.83, 603.11, 584.64, 565.67, 526.69, 474.55, 425.56.

Sodium (*E*)-2-(((4-hydroxyphenethyl)imino)methyl)benzenesulfonate (11)

Tyramine (137 mg, 1 mmol) and sodium 2-formylbenzenesulfonate (208 mg, 1 mmol) were taken to reaction. Product was obtained with 97% yield (318 mg, mp 143-145 °C).

^{1}**H NMR** (700 MHz, D_2O) δ ppm 2.86 (t, *J*=6.99 Hz, 2 H), 3.79 (td, *J*=6.99, 1.08 Hz, 2 H), 6.71 (m, 2 H), 7.05 - 7.10 (m, 2 H), 7.50 (m, 2 H), 7.69 (m, 1 H), 7.79 - 7.81 (m, 1 H), 8.81 (s, 1 H).

^{13}**C NMR** (176 MHz, D_2O) δ ppm 35.30, 61.75, 115.83, 126.76, 128.15, 130.39, 130.75, 131.71, 132.50, 134.49, 141.60, 155.26, 162.18.

HRMS (ESI-TOF) *m/z*: $[M - Na]^-$ Calcd for: $C_{15}H_{14}NNaO_4S$ Molecular Weight: 304.0644; found 304.0605.

FTIR (ATR) (cm^{-1}): 3302.83, 2916.26, 2855.84, 2167.87, 2127.56, 2026.72, 1998.34, 1636.26, 1613.08, 1595.38, 1569.11, 1514.28, 1443.23, 1374.31, 1181.83, 1137.93, 1105.76, 1088.89, 1019.59, 966.36, 824.99, 761.31, 737.69, 718.47, 614.17, 565.91, 508.45, 468.94, 442.04, 421.98.

Sodium (*E*)-2-(((2-(1H-imidazol-4-yl)ethyl)imino)methyl)benzenesulfonate (12)

Histamine (111 mg, 1 mmol) and sodium 2-formylbenzenesulfonate (208 mg, 1 mmol) were taken to reaction. Product was obtained with 97% yield (292 mg, mp 169-170 °C).

^{1}**H NMR** (700 MHz, D_2O) δ ppm 2.92 (t, *J*=6.78 Hz, 2 H), 3.83 (td, *J*=6.78, 1.08 Hz, 2 H), 7.48 - 7.54 (m, 2 H), 7.55 - 7.57 (m, 1 H), 7.71 (m, 1 H), 7.80 - 7.83 (m, 1 H), 7.91 (m, 1H), 8.85 (s, 1 H).

^{13}**C NMR** (176 MHz, D_2O) δ ppm 27.21, 59.60, 123.07, 126.79, 128.13, 130.85, 131.72, 131.88, 132.39, 135.78, 141.65, 162.47.

HRMS (ESI-TOF) *m/z*: $[M + H]^+$ Calcd for: $C_{12}H_{12}N_3NaO_3S$ Molecular Weight: 302.0575; found 302.0488.

FTIR (ATR) (cm^{-1}): 3218.72, 2916.95, 2851.50, 2325.60, 2202.00, 2167.46, 2106.56, 1634.73, 1567.30, 1465.66, 1439.93, 1381.65, 1184.06, 1137.08, 1086.91, 1019.46, 938.91, 818.72, 761.82, 722.73, 660.53, 612.84, 566.68, 517.33, 468.74, 437.18.

Sodium 2,2'-((1*E*,1'*E*)-(butane-1,4-diylbis(azanylylidene))bis(methanylylidene))dibenzene-sulfonate (13)

Putrescine (88 mg, 1 mmol) and sodium 2-formylbenzenesulfonate (416 mg, 2 mmol) were taken to reaction. Product was obtained with 98% yield (459 mg, mp 187-189 °C).

^{1}H NMR (700 MHz, D_2O) δ ppm 1.69 - 1.73 (m, 4 H), 3.63 - 3.68 (m, 4 H), 7.50 - 7.58 (m, 4 H), 7.78 (m, 2 H), 7.83 - 7.87 (m, 2 H), 9.00 (s, 1 H), 9.02 (s, 1 H).

^{13}C NMR (101 MHz, D_2O) δ ppm 28.93, 60.12, 126.80, 128.11, 130.76, 131.77, 132.57, 141.69, 161.88.

HRMS (ESI-TOF) *m/z*: $[M + H]^+$ Calcd for: $C_{18}H_{18}N_2Na_2O_6S_2$ Molecular Weight: 469.0480; found 469.0379.

FTIR (ATR) (cm^{-1}): 3337.41, 3066.40, 2931.10, 2852.69, 2110.43, 1634.99, 1568.24, 1410.50, 1332.05, 1216.62, 1187.75, 1125.18, 1091.73, 1041.13, 1021.12, 995.40, 956.22, 923.40, 820.33, 802.48, 749.87, 714.75, 689.49, 648.34, 610.86, 567.31, 532.43, 513.63, 462.85, 438.00.

Sodium 2,2'-((1*E*,1'*E*)-(pentane-1,5-diylbis(azanylylidene))bis(methanylylidene))dibenzene-sulfonate (14)

Cadaverine (102 mg, 1 mmol) and sodium 2-formylbenzenesulfonate (416 mg, 2 mmol) were taken to reaction. Product was obtained with 97% yield (468 mg, mp 188-190 °C).

^{1}H NMR (700 MHz, D_2O) δ ppm 1.38 - 1.44 (m, 2 H), 1.61 - 1.73 (m, 4 H), 3.60 (q, *J*=7.03 Hz, 4 H), 7.42 (m, 1 H), 7.47 (m, 1 H), 7.51 - 7.57 (m, 2 H), 7.66 (m, 1 H), 7.77 (m, 1 H), 7.81 (m, 1 H), 7.84 - 7.86 (m, 1 H), 8.97 (s, 1 H), 8.99 (s, 1 H).

^{13}C NMR (176 MHz, D_2O) δ ppm 23.64, 29.38, 60.31, 126.77, 126.84, 128.07, 128.10, 130.69, 130.77, 131.71, 131.76, 132.54, 132.61, 141.67, 141.75, 161.69, 161.73.

HRMS (ESI-TOF) *m/z*: $[M - 2Na]^{2-}$ Calcd for: $C_{19}H_{20}N_2Na_2O_6S_2$ Molecular Weight: 437.0763; found 437.0863.

FTIR (ATR) (cm^{-1}): 3362.13, 3065.02, 2931.50, 2853.47, 2112.86, 1634.59, 1567.90, 1411.26, 1332.58, 1186.74, 1126.04, 1090.27, 1041.23, 1019.60, 995.63, 956.41, 923.52, 820.53, 802.50, 761.77, 749.79, 715.18, 689.44, 648.12, 610.43, 566.77, 532.83, 513.76, 461.36, 435.91.

Sodium 2,2'-((1*E*,15*E*)-2,6,11,15-tetraazahexadeca-1,15-diene-1,16-diyl)dibenzenesulfonate (15)

Spermine (202 mg, 1 mmol) and sodium 2-formylbenzenesulfonate (416 mg, 2 mmol) were taken to reaction. Product was obtained with 95% yield (554 mg, mp 170-172 °C).

^{1}H NMR (700 MHz, D_2O) δ ppm 1.06 - 1.23 (m, 2 H), 2.08 - 2.21 (m, 2 H), 2.52 - 2.57 (m, 2 H), 2.95 (m, 2 H), 3.54 - 3.64 (m, 2 H), 7.32 - 7.44 (m, 2 H), 7.54 (m, 2H), 7.74 - 7.88 (m, 4 H), 8.99 (s, 2 H).

^{13}C NMR (176 MHz, D_2O) δ ppm 25.28, 29.09, 46.24, 47.89, 60.13, 127.11, 128.50, 130.91, 131.79, 138.40, 141.85, 162.21.

HRMS (ESI-TOF) *m/z*: $[M - 2Na]^{2-}$ Calcd for: $C_{24}H_{32}N_4Na_2O_6S_2$ Molecular Weight: 537.1763; found 537.1857.

FTIR (ATR) (cm^{-1}): 3406.46, 3275.56, 2936.08, 2849.88, 2113.60, 1637.34, 1556.95, 1408.32, 1181.33, 1112.60, 1033.97, 985.87, 924.76, 800.50, 726.11, 693.44, 645.77, 615.66, 513.62.

Sodium 2-((*E*)-((3-((4-(((E)-2-sulfonatobenzylidene)amino)butyl)amino)propyl)imino)methyl) benzenesulfonate (16)

Spermidine (145 mg, 1 mmol) and sodium 2-formylbenzenesulfonate (416 mg, 2 mmol) were taken to reaction. Product was obtained with 98% yield (515 mg, mp 196-198 °C).

^{1}H NMR (700 MHz, D_2O) δ ppm 1.33 - 1.50 (m, 4 H), 1.64 (m, 2 H), 2.19 - 2.32 (m, 2 H), 2.49 - 2.64 (m, 2 H), 3.42 (m, 2 H), 3.58-3.62 (m, 2 H), 7.41 (m, 1 H), 7.47 - 7.59 (m, 2 H), 7.64 (m, 1 H), 7.62 (m, 1 H), 7.74 (m, 1 H), 7.84 (m, 1 H), 8.78 (s, 1 H), 8.98 (s, 1 H).

^{13}C NMR (176 MHz, D_2O) δ ppm 23.49, 25.36, 27.50, 48.96, 53.00, 53.83, 59.98, 126.79, 127.08, 127.88, 128.27, 128.38, 130.72, 131.78, 132.02, 12.56, 138.38, 141.12, 141.67, 161.68, 161.88.

HRMS (ESI-TOF) *m/z*: $[M + H]^+$ Calcd for: $C_{21}H_{26}N_3Na_2O_6S_2$ Molecular Weight: 526.1058; found 526.0891.

FTIR (ATR) (cm^{-1}): 3425.71, 3073.16, 3055.65, 2923.55, 2849.87, 2830.45, 2091.18, 1644.76, 1576.21, 1547.15, 1454.84, 1420.48, 1357.12, 1335.10, 1279.67, 1233.19, 1184.09, 1125.91, 1085.58, 1020.90, 995.00, 957.95, 932.87, 903.66, 869.88, 814.57, 794.84, 766.06, 731.47, 687.55, 655.52, 615.28, 565.38, 526.33, 481.18, 424.04.

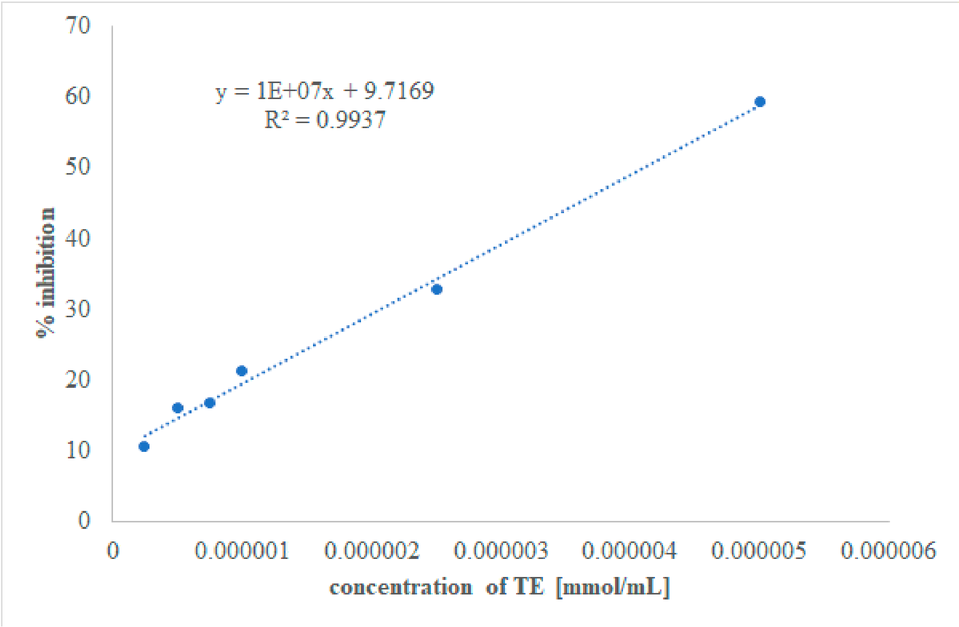

Figure S1. The calibration curve for ABTS assay

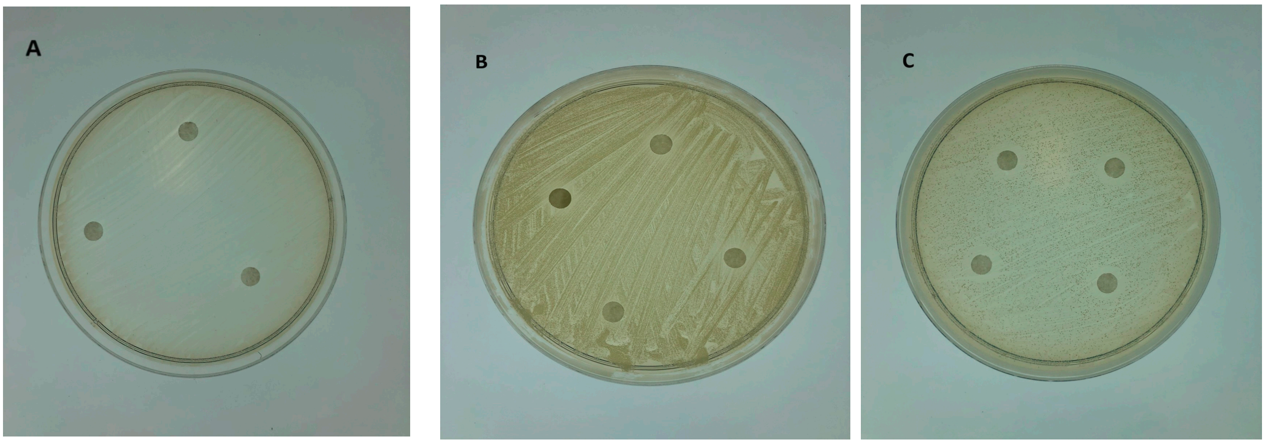

Figure S2. The inhibition zones of compounds **7** for *E. coli*; where (A) concentration 0.01 g·mL^{-1}; (B) concentration 0.02 g·mL^{-1}; (C) concentration 0.04 g·mL^{-1}

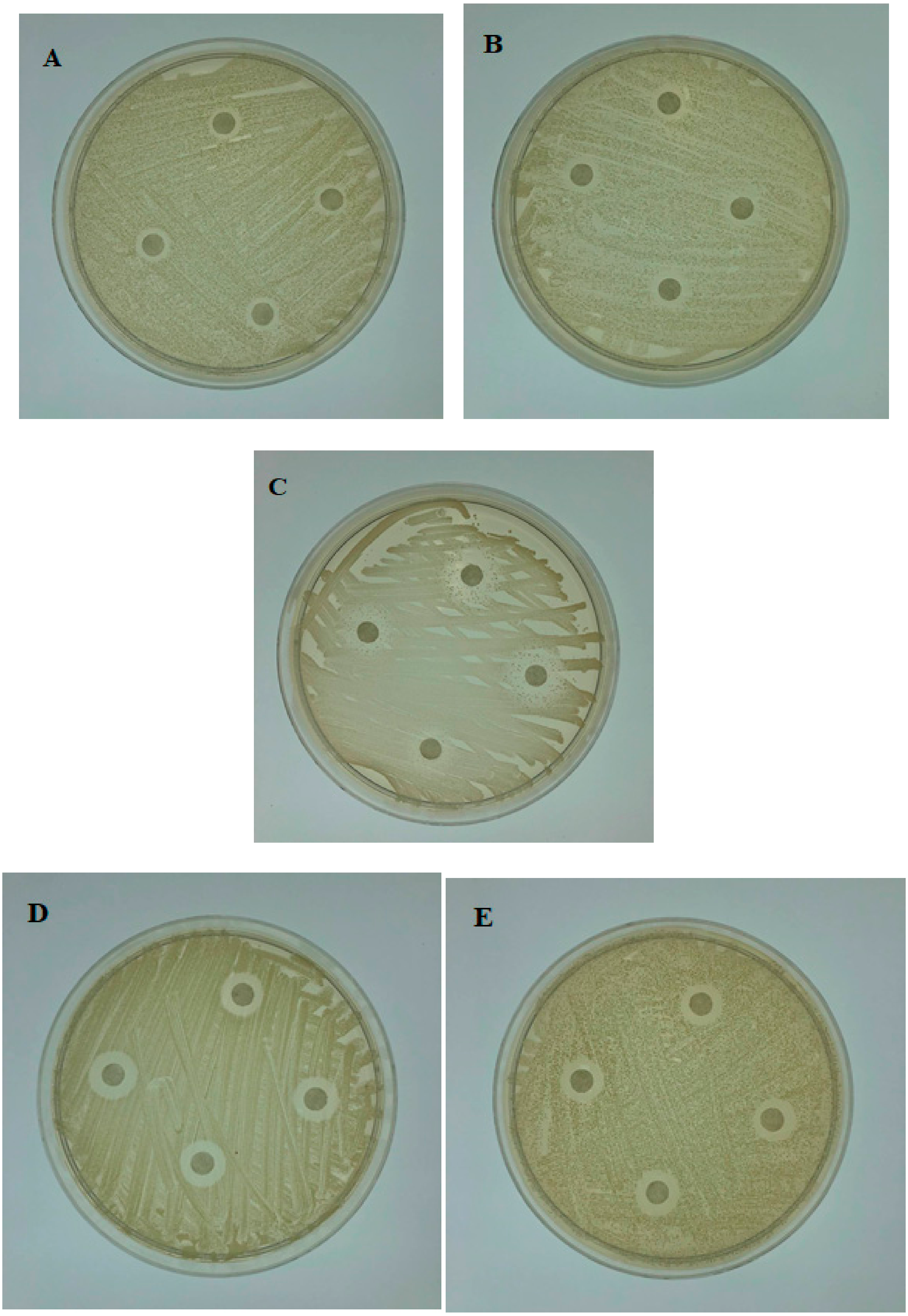

Figure S3. The inhibition zones of compounds: **9** for *E. coli* (A); **1** for *S. aureus* (B); **9** for *S. aureus* (C), **Phen** for *E. coli* (D) and *S. aureus* (E)

^{1}H and ^{13}C NMR, ESI-MS spectras

Sodium (*E*)-3-((phenethylimino)methyl)benzenesulfonate (1)

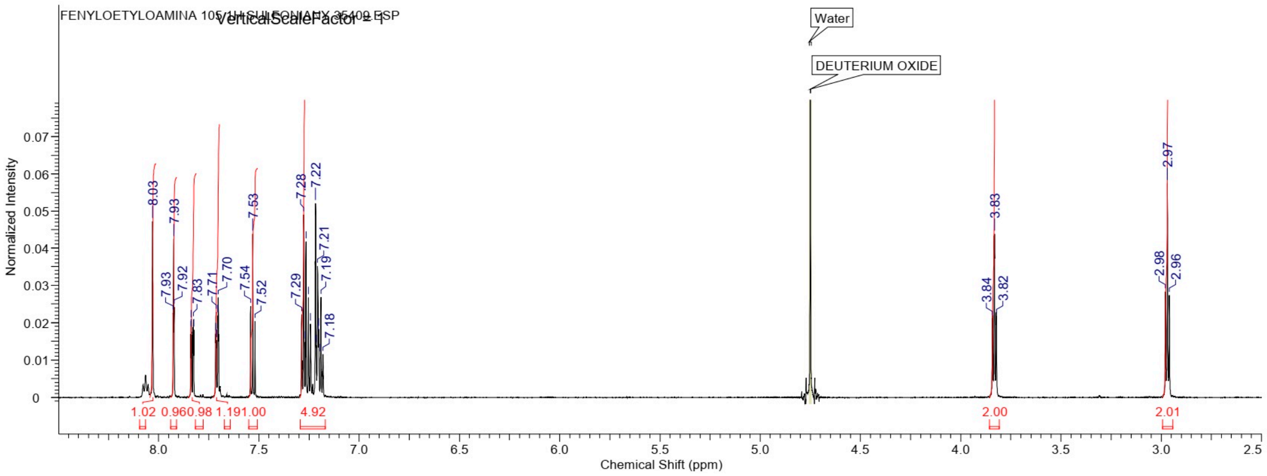

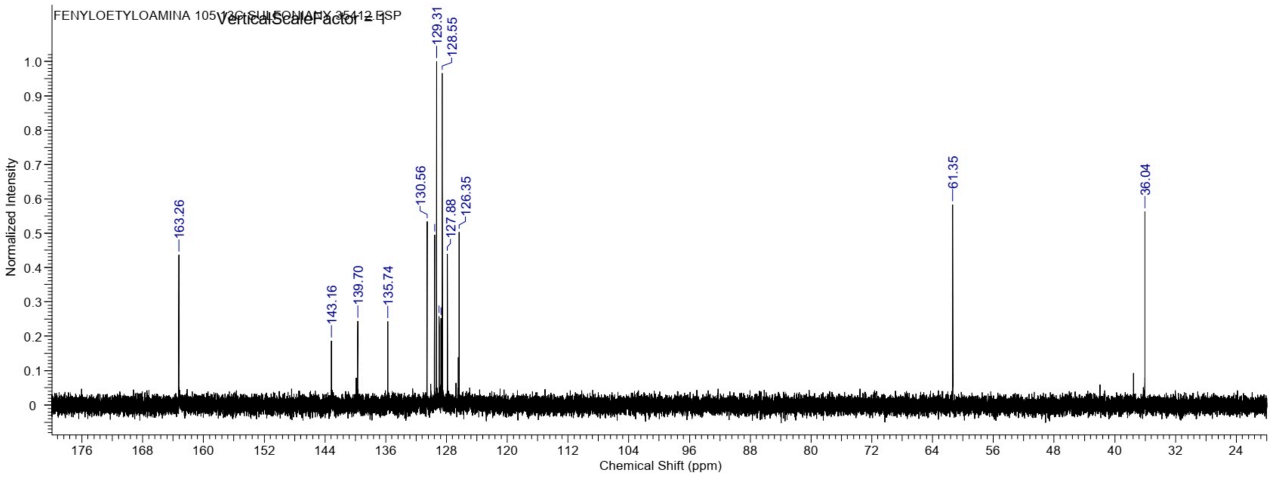

ESI+

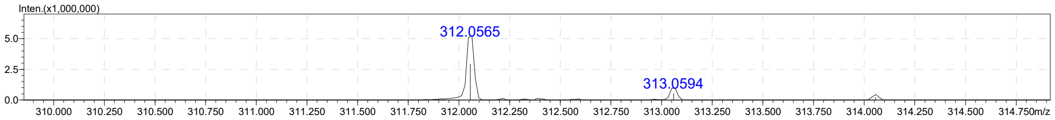

Sodium (*E*)-3-(((2-(1*H*-indol-3-yl)ethyl)imino)methyl)benzenesulfonate (2)

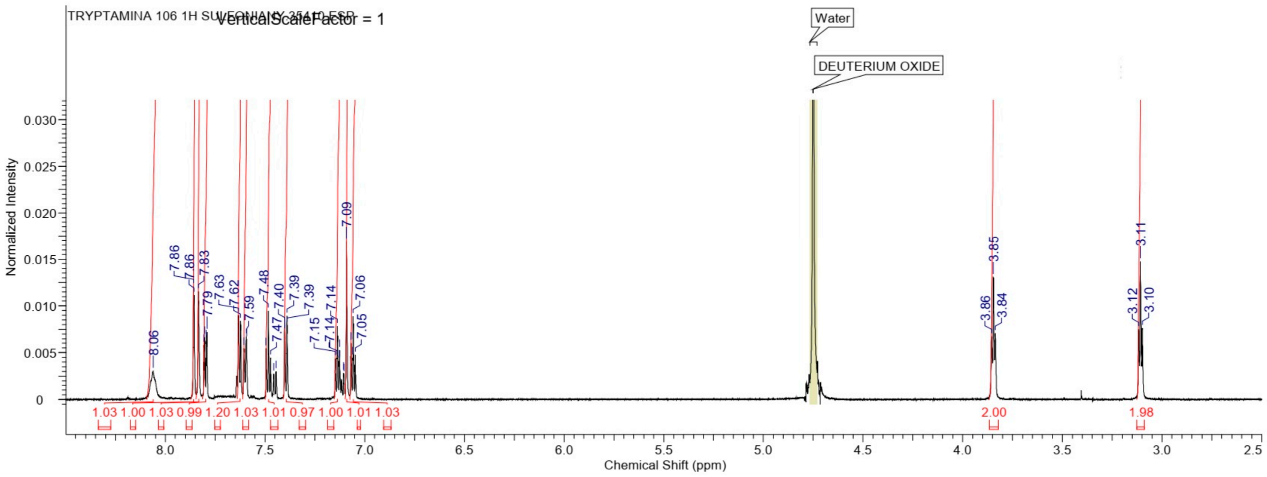

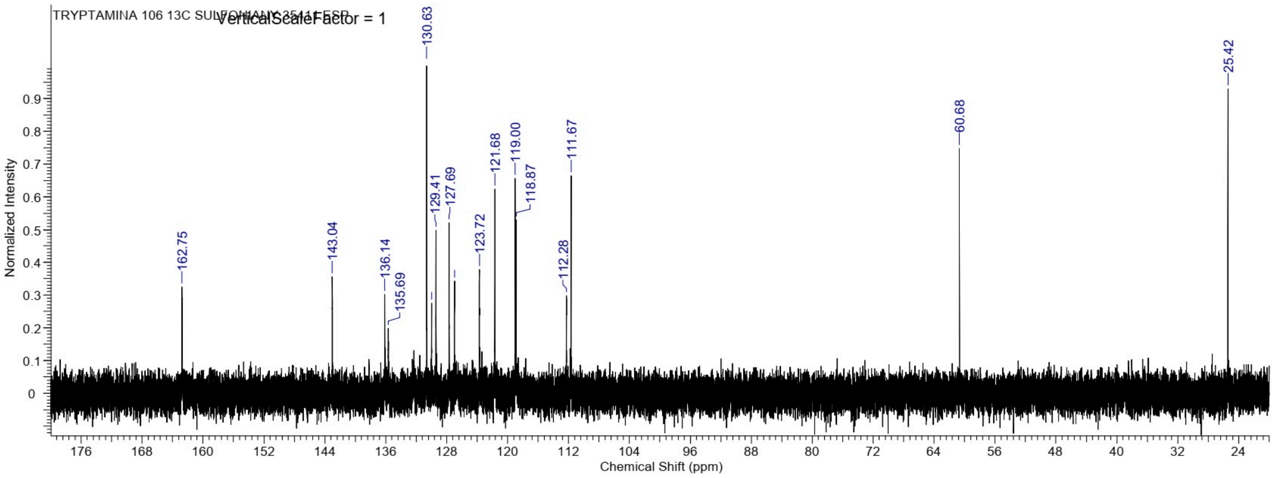

ESI-

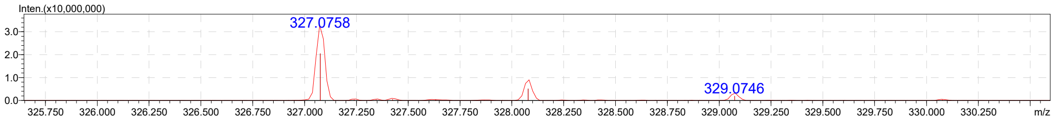

Sodium (*E*)-3-(((4-methylphenethyl)imino)methyl)benzenesulfonate (3)

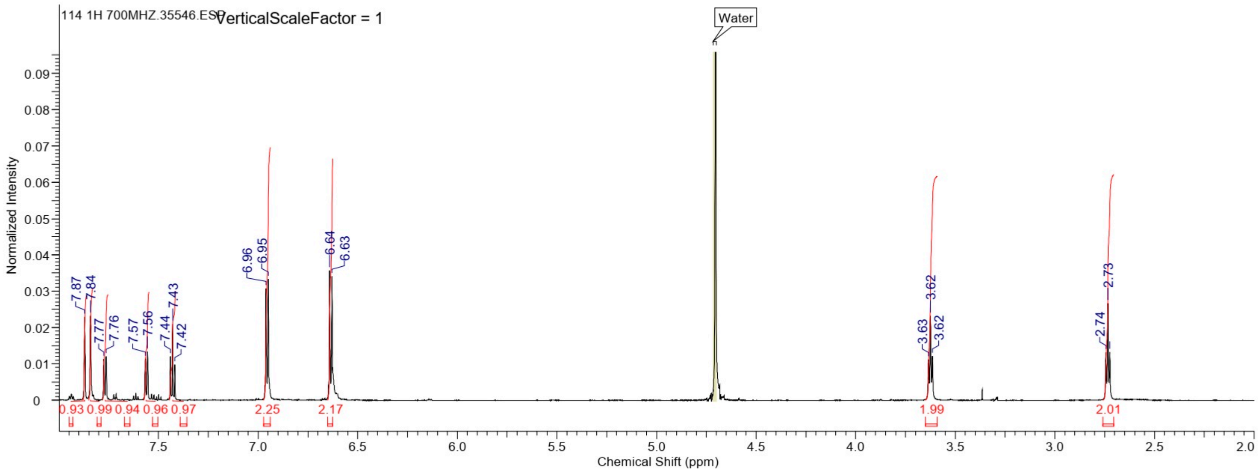

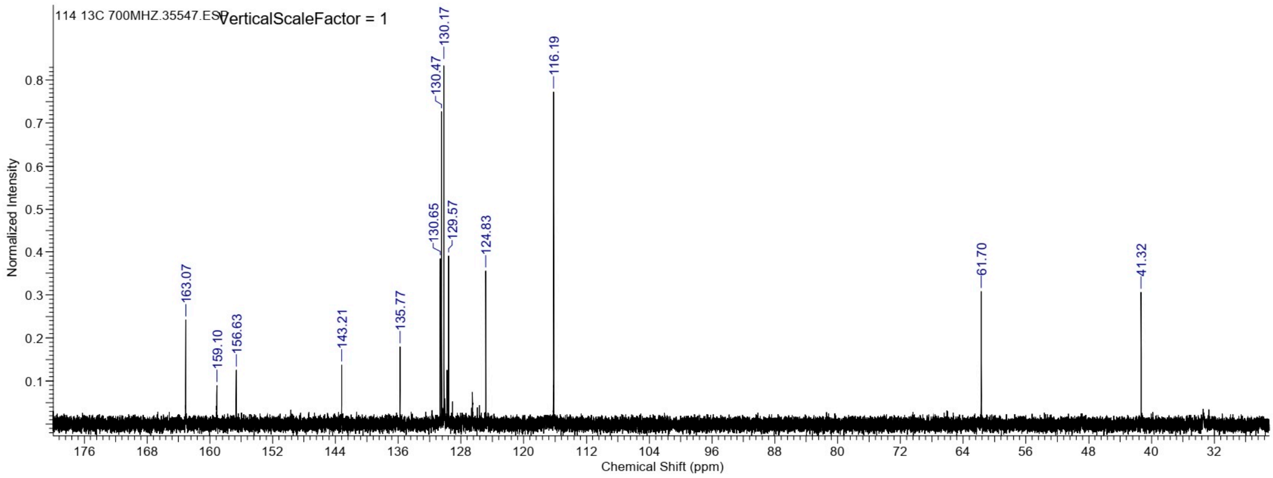

ESI-

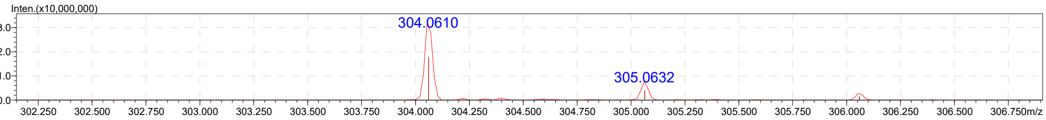

Sodium (E)-3-(((2-(1H-imidazol-4-yl)ethyl)imino)methyl)benzenesulfonate (4)

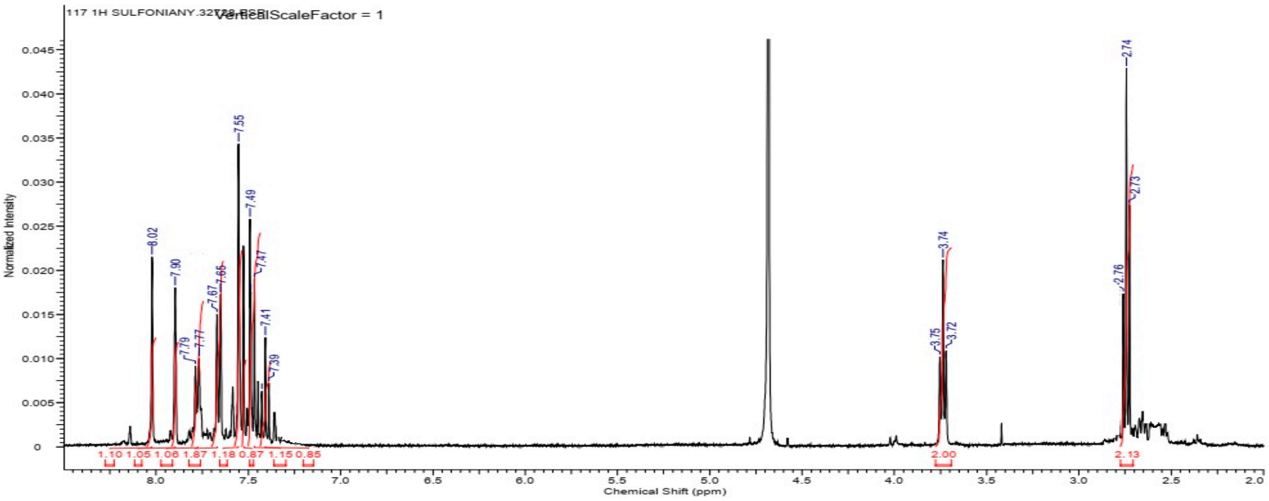

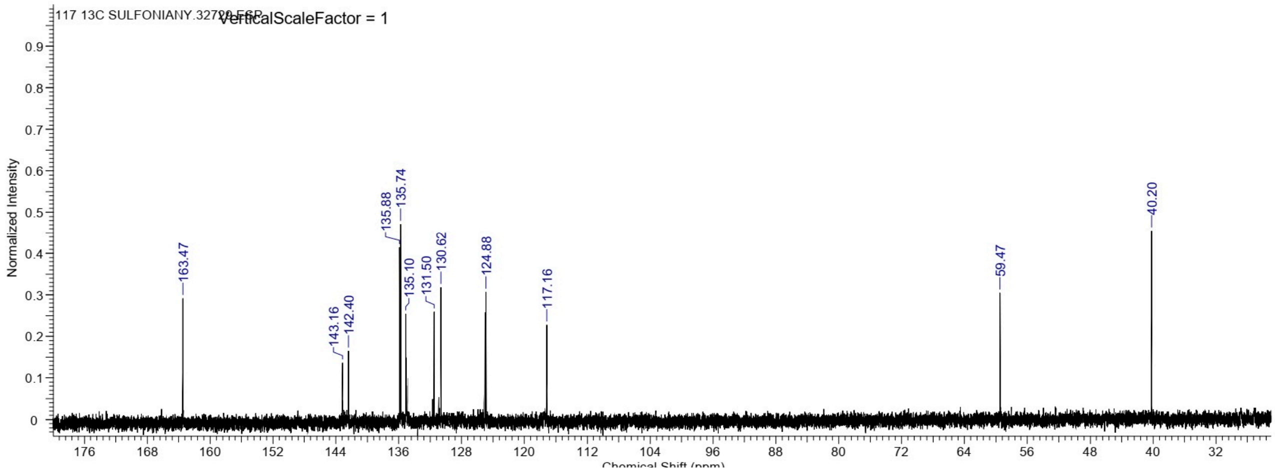

ESI+

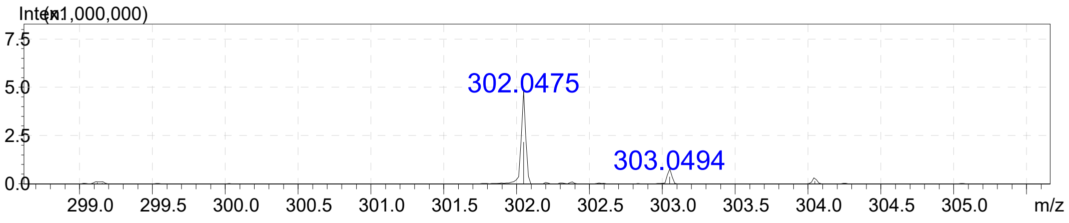

Sodium 3,3'-((1E,1'E)-(butane-1,4-diylbis(azanylylidene))bis(methanylylidene))dibenzene-sulfonate (5)

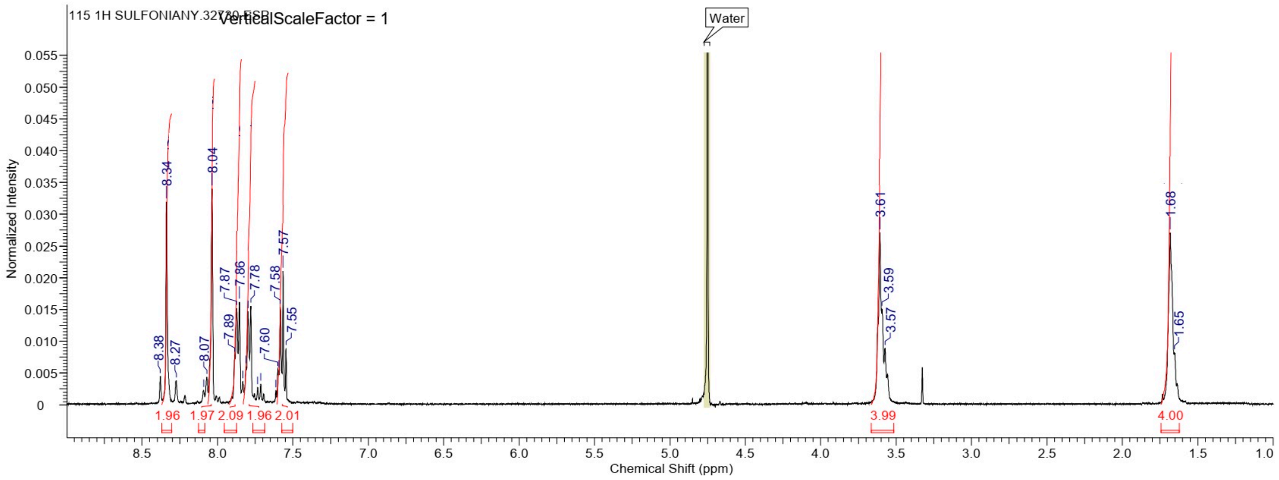

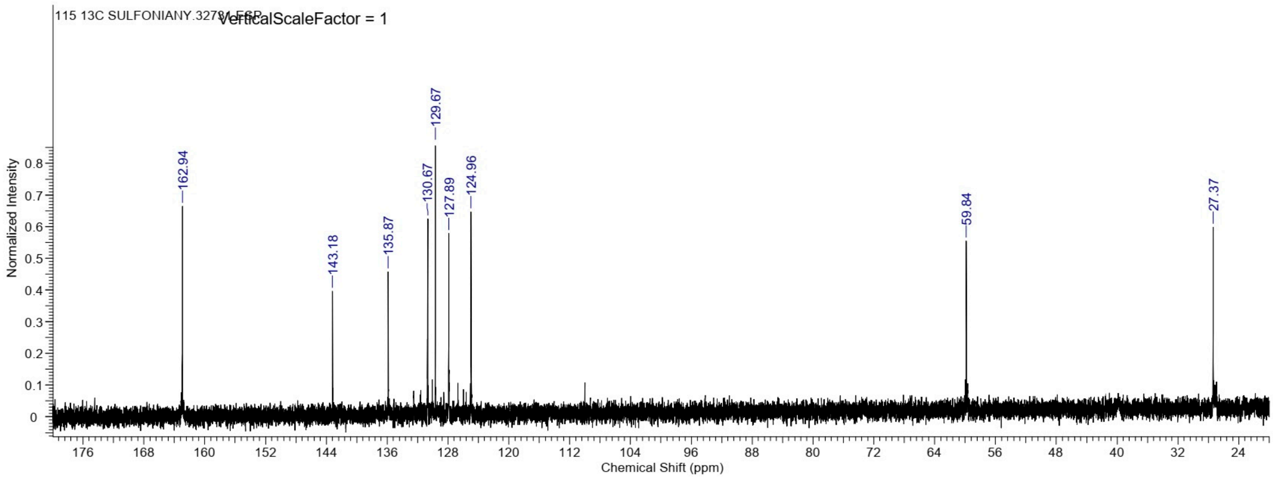

ESI-

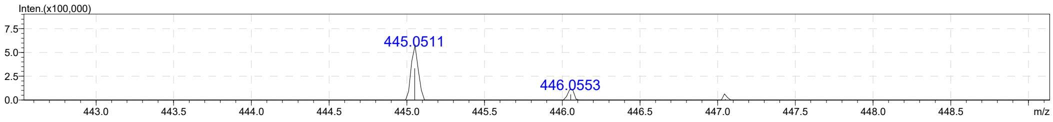

Sodium 3,3'-((1E,1'E)-(pentane-1,5-diylbis(azanylylidene))bis(methanylylidene))dibenzene-sulfonate (6)

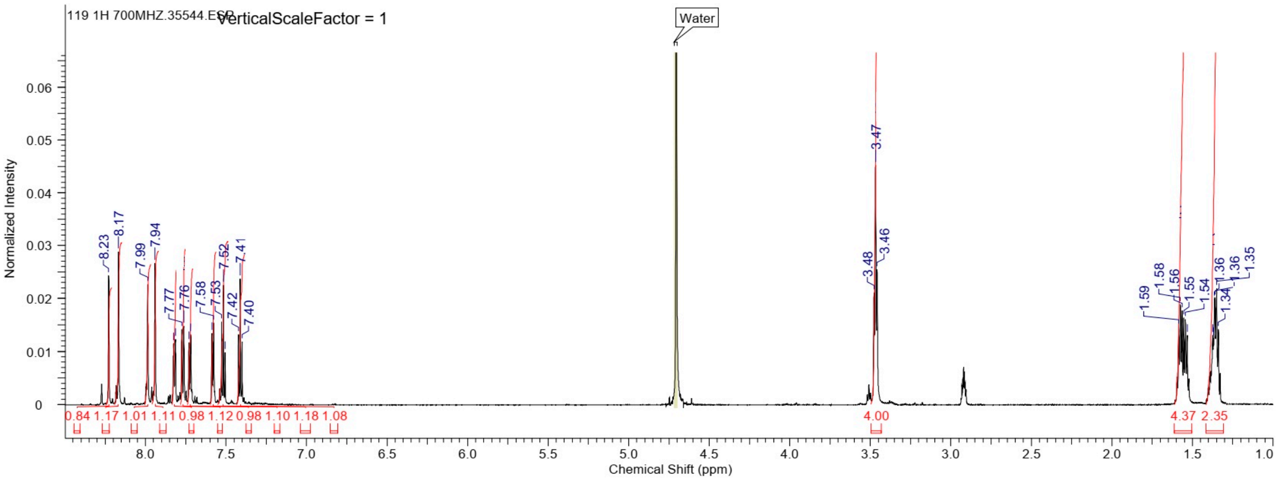

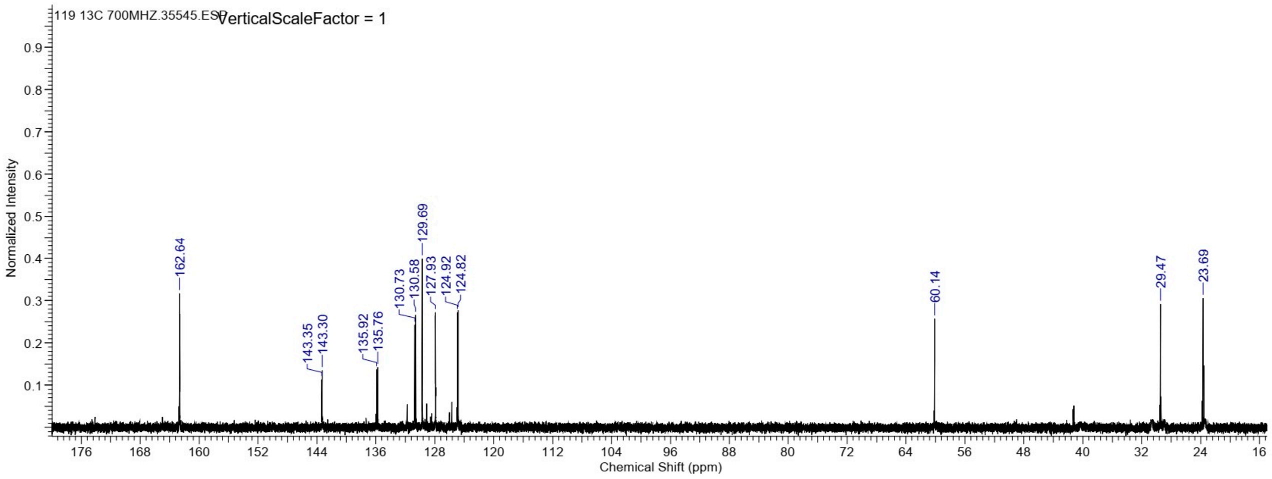

ESI-

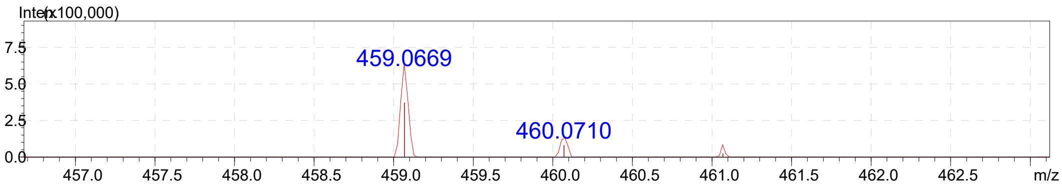

Sodium 3,3'-((1E,15E)-2,6,11,15-tetraazahexadeca-1,15-diene-1,16-diyl)dibenzenesulfonate (7)

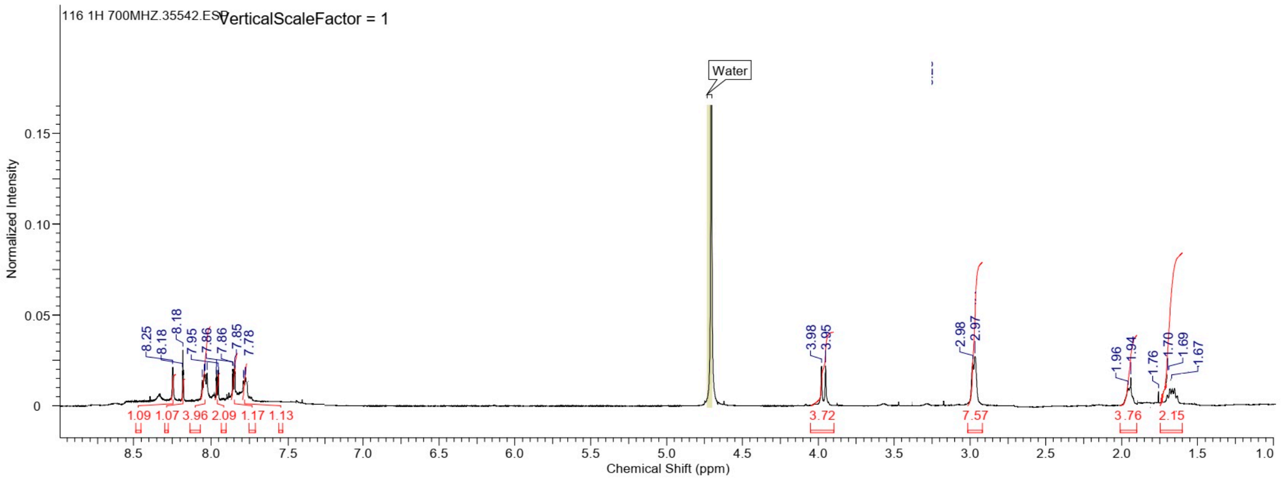

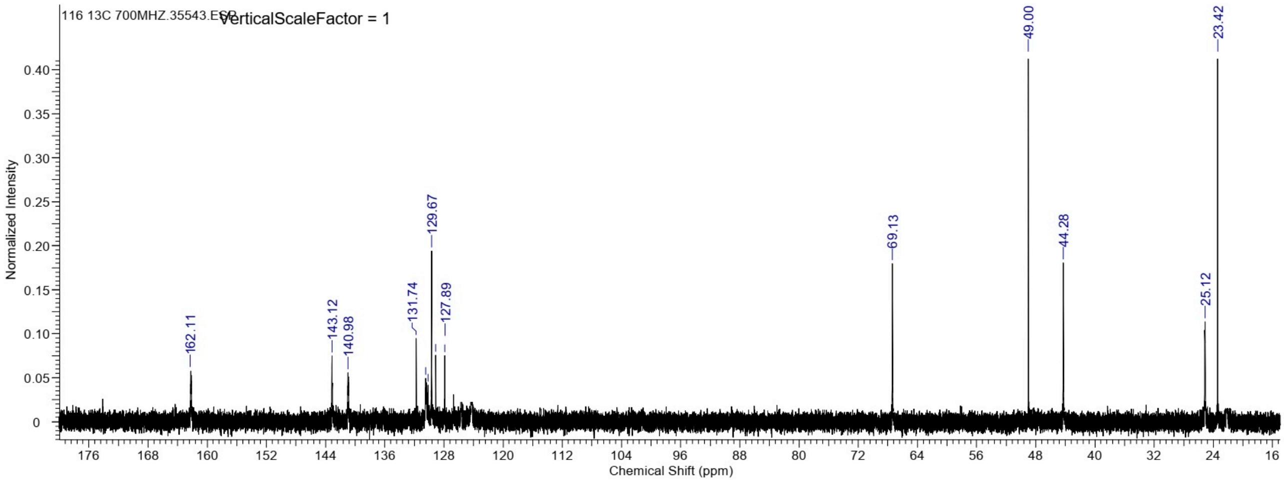

ESI+

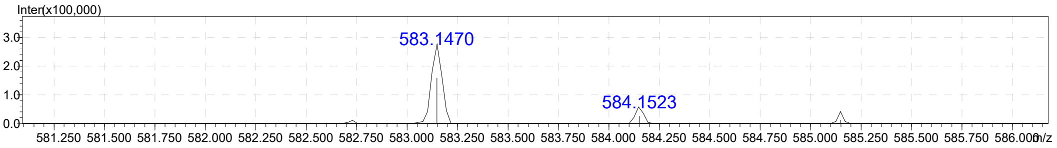

Sodium 3-((E)-((3-((4-(((E)-3-sulfonatobenzylidene)amino)butyl)amino)propyl)imino)methyl) benzenesulfonate (8)

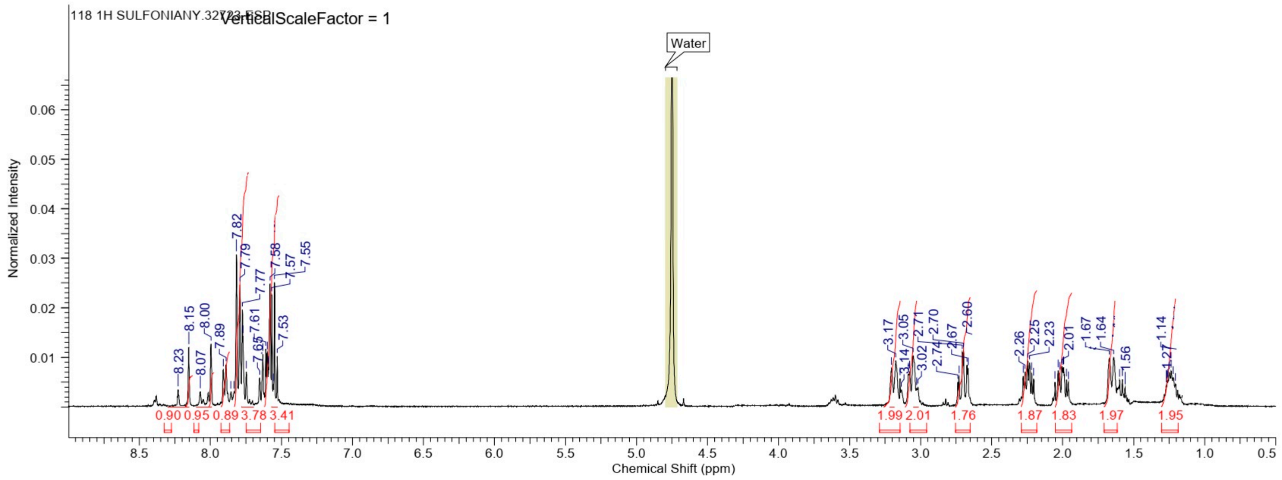

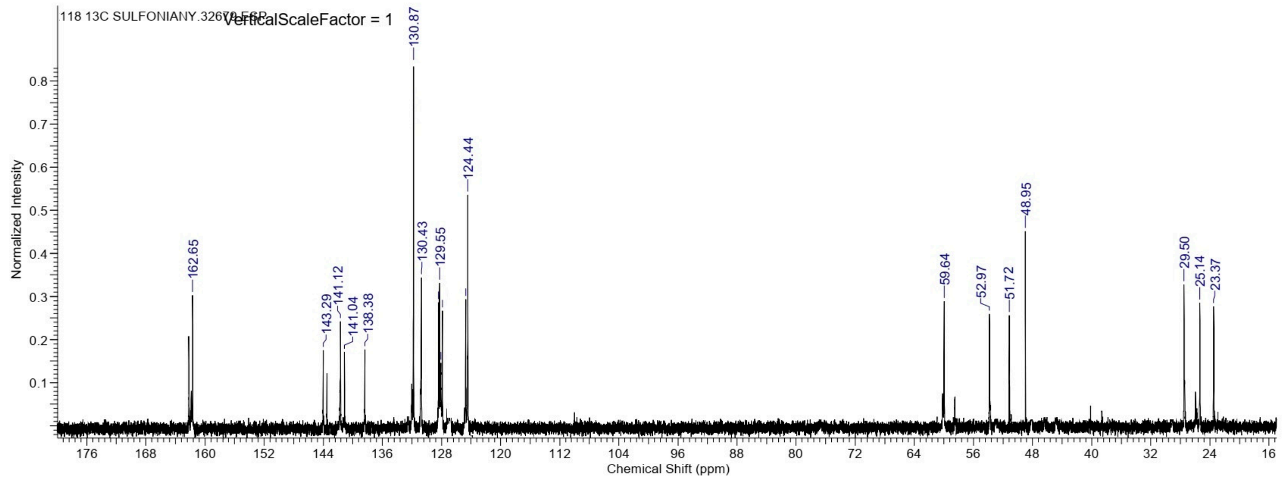

ESI+

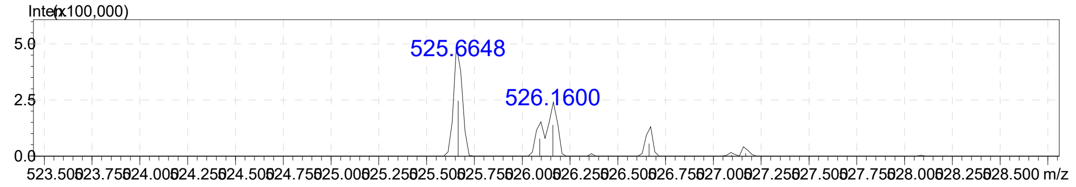

Sodium (E)-2-((phenethylimino)methyl)benzenesulfonate (9)

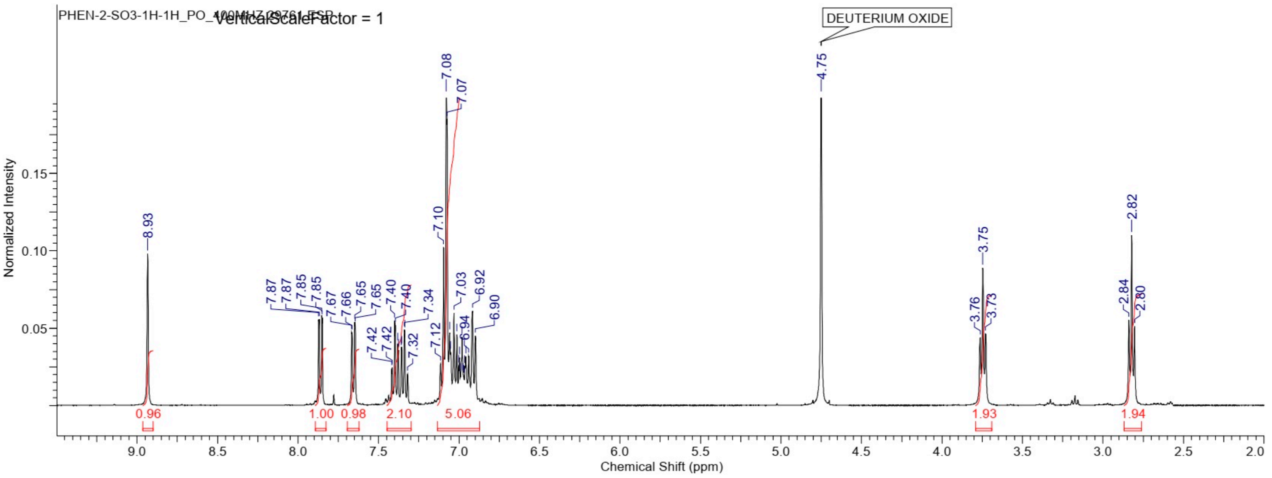

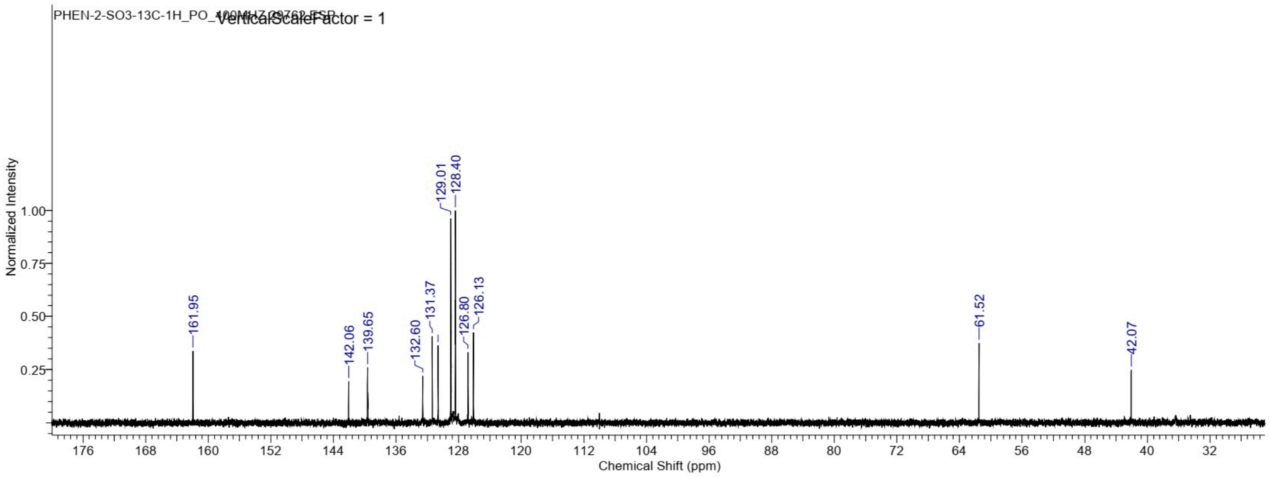

ESI+

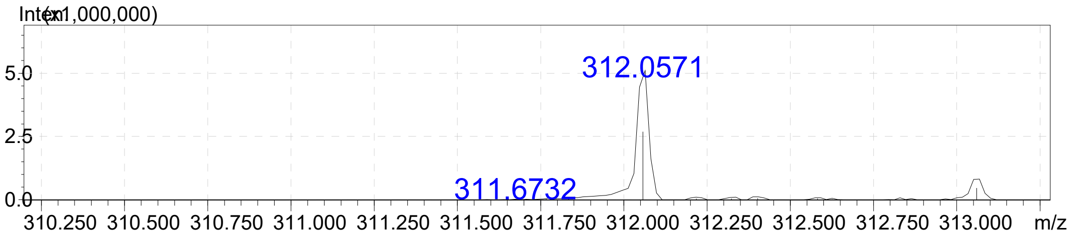

Sodium (E)-2-(((2-(1H-indol-3-yl)ethyl)imino)methyl)benzenesulfonate (10)

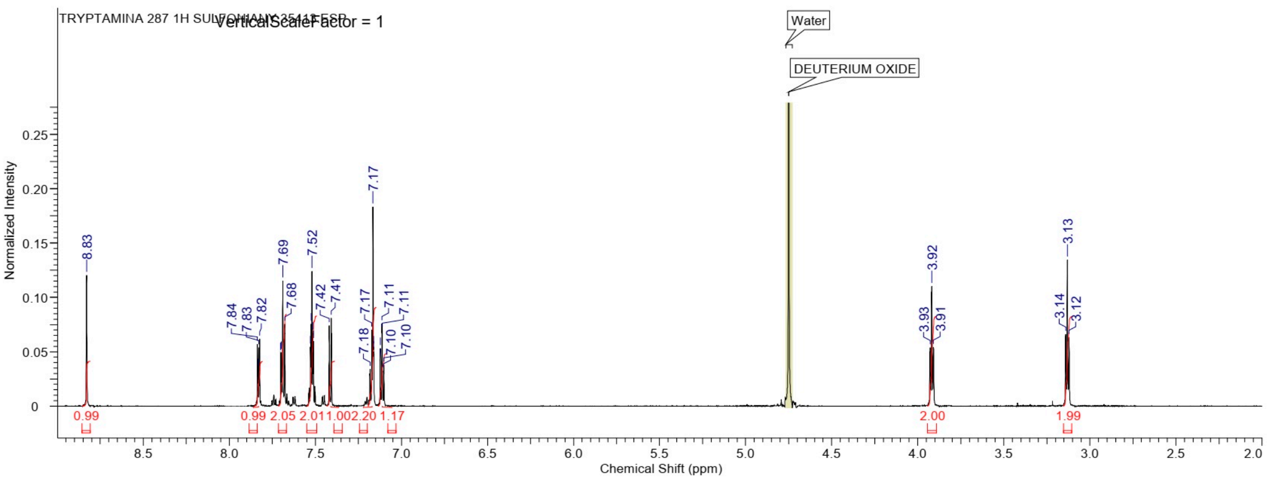

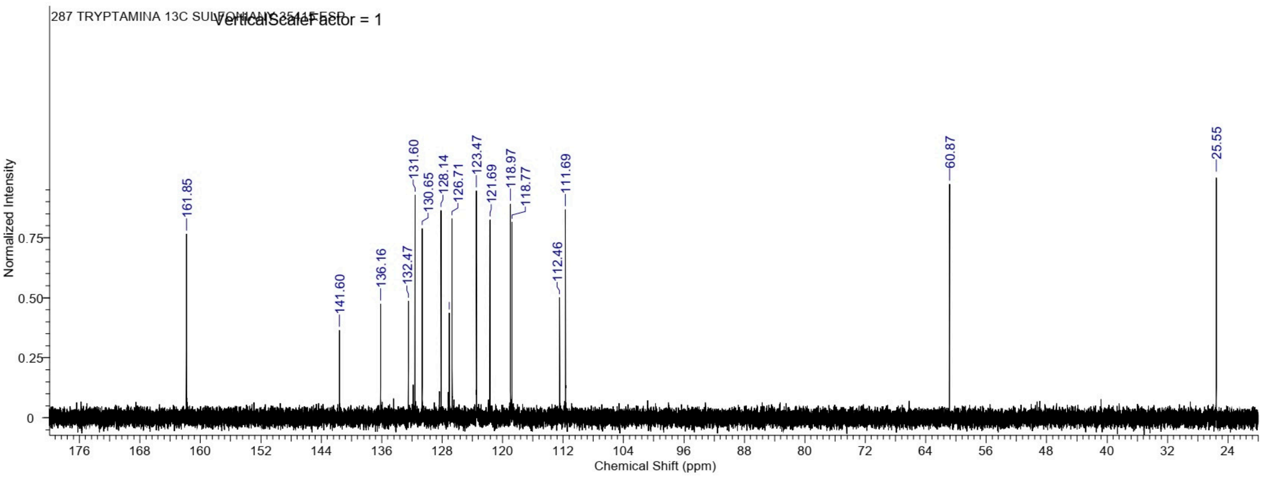

ESI-

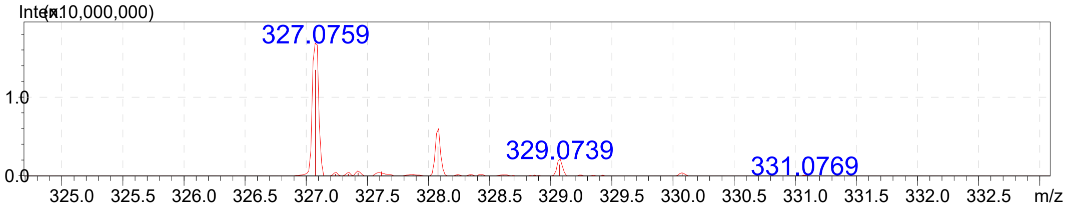

Sodium (E)-2-(((4-hydroxyphenethyl)imino)methyl)benzenesulfonate (11)

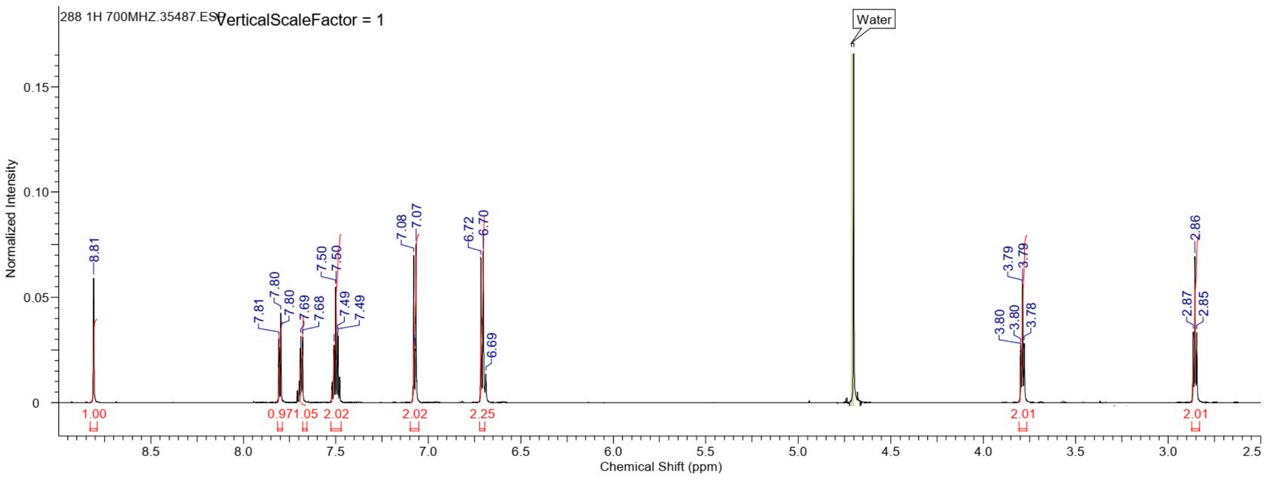

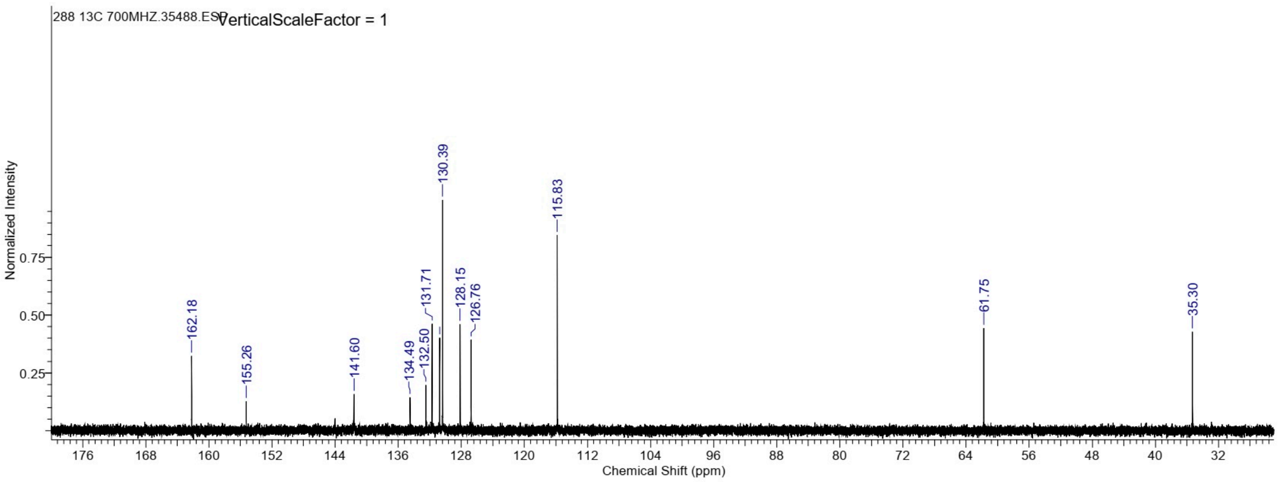

ESI-

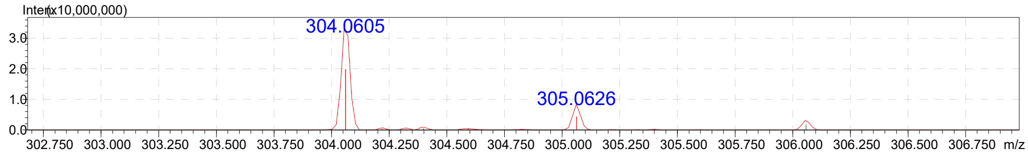

Sodium (E)-2-(((2-(1H-imidazol-4-yl)ethyl)imino)methyl)benzenesulfonate (12)

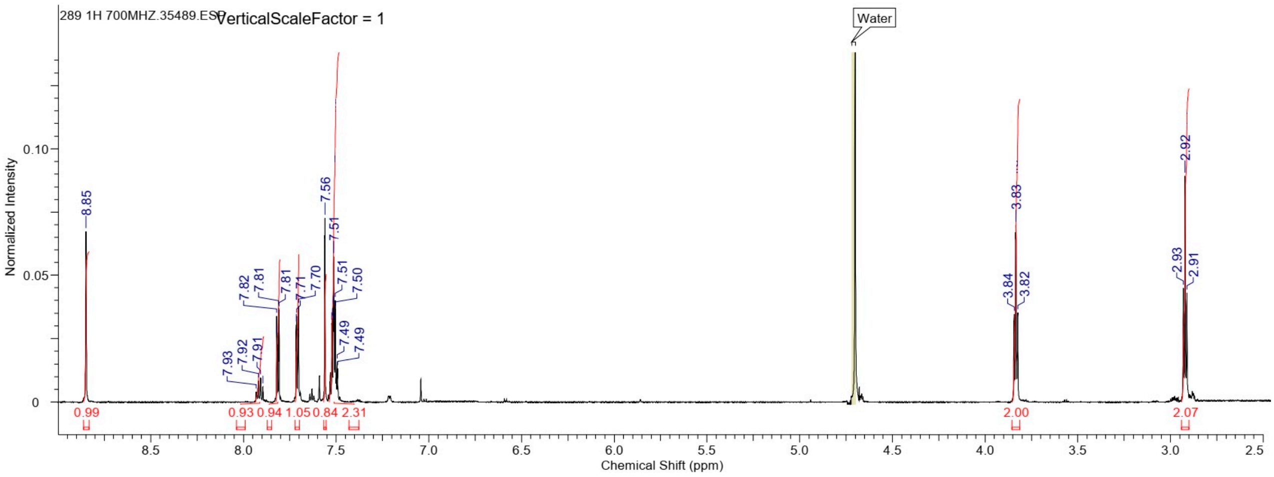

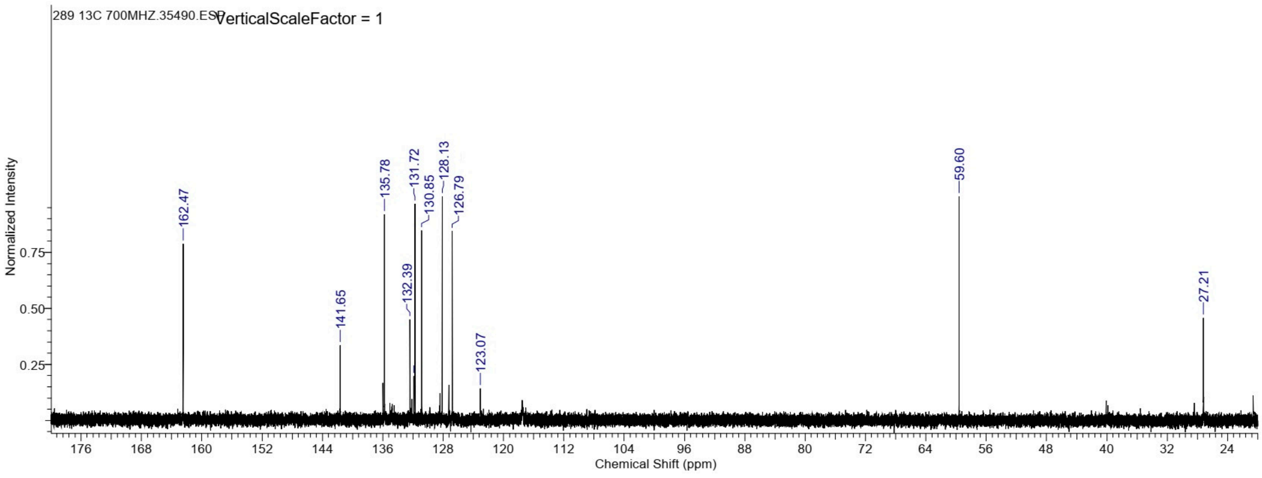

ESI+

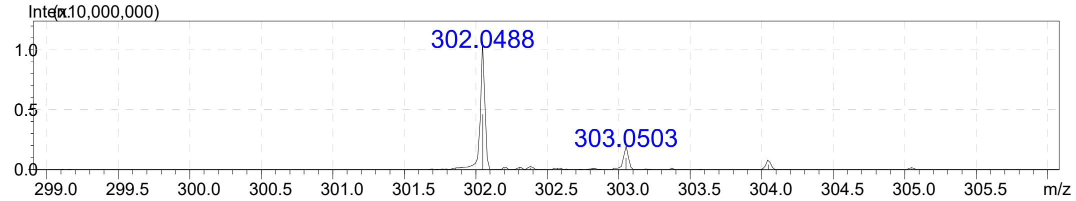

Sodium 2,2'-((1E,1'E)-(butane-1,4-diylbis(azanylylidene))bis(methanylylidene))dibenzene-sulfonate (13)

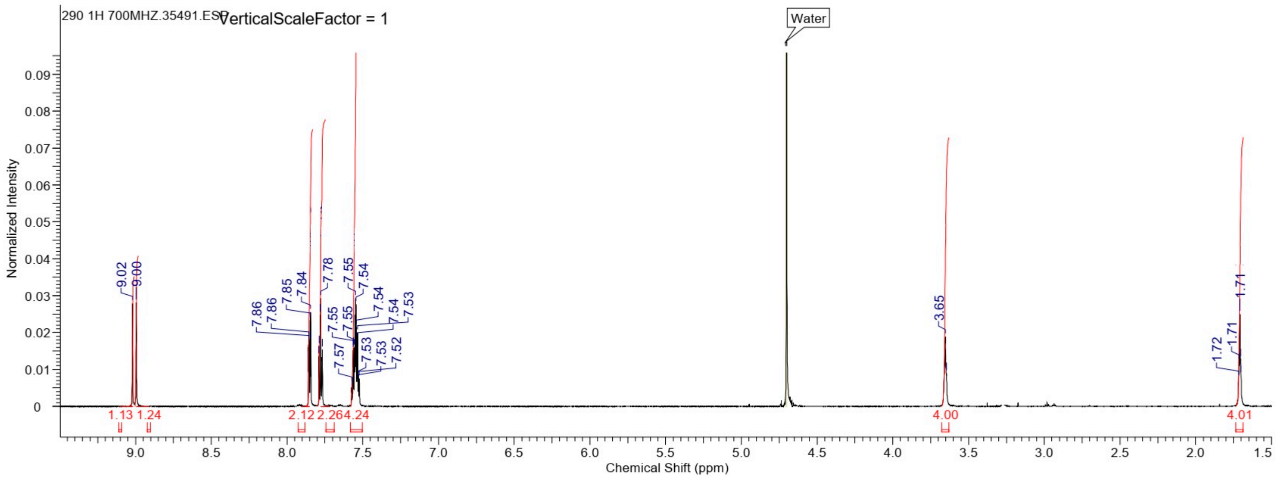

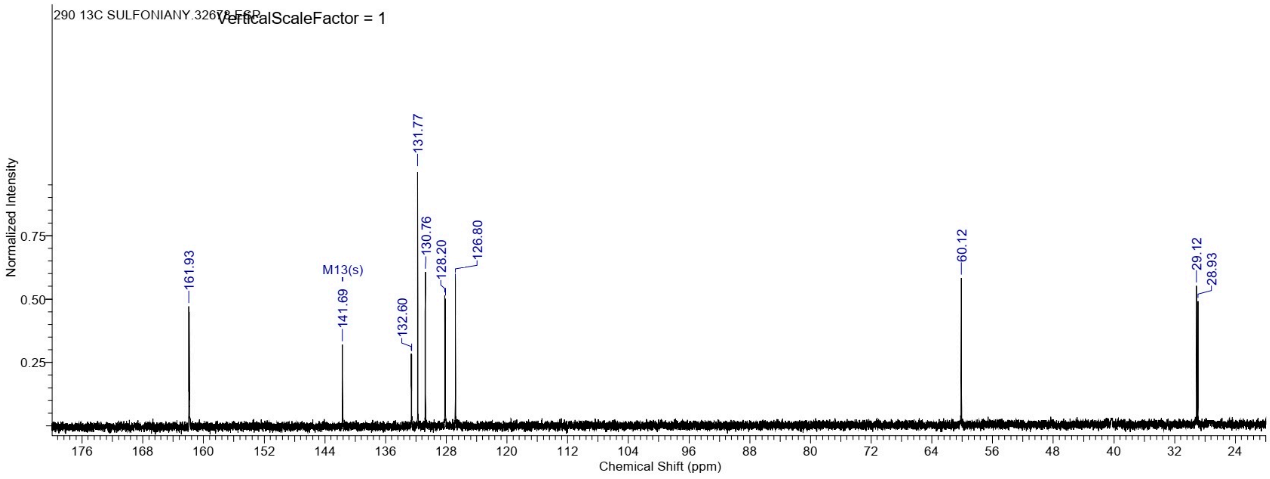

ESI+

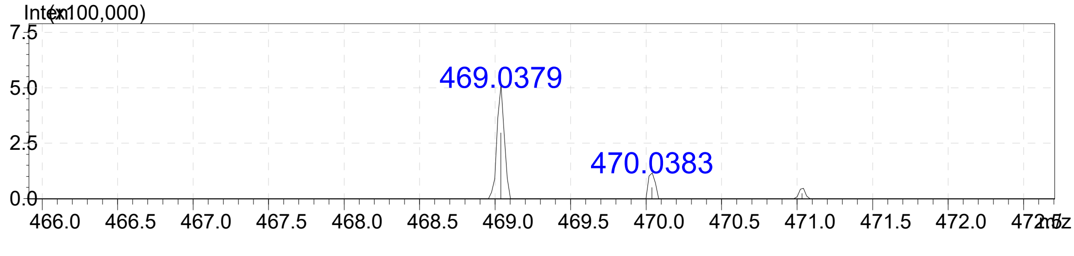

Sodium 2,2'-((1E,1'E)-(pentane-1,5-diylbis(azanylylidene))bis(methanylylidene))dibenzene-sulfonate (14)

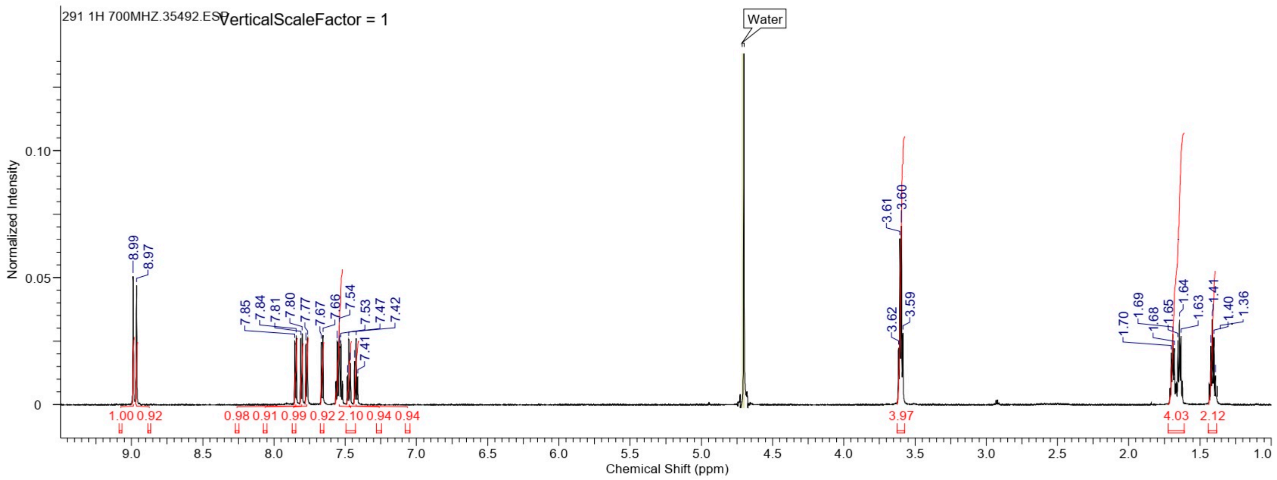

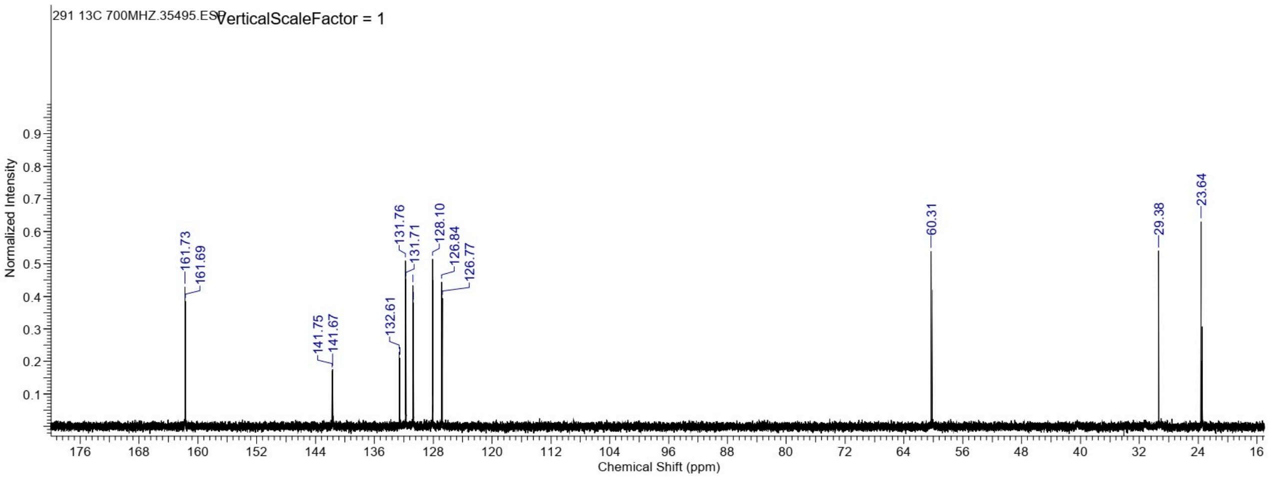

ESI-

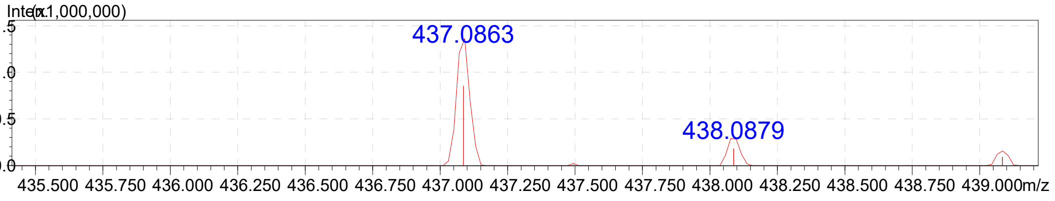

Sodium 2,2'-((1E,15E)-2,6,11,15-tetraazahexadeca-1,15-diene-1,16-diyl)dibenzenesulfonate (15)

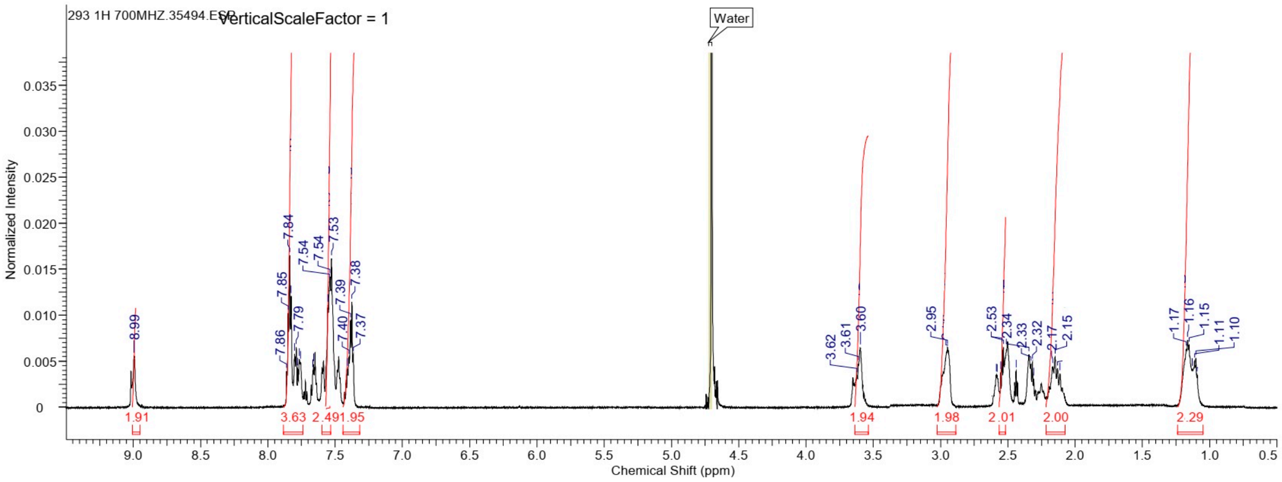

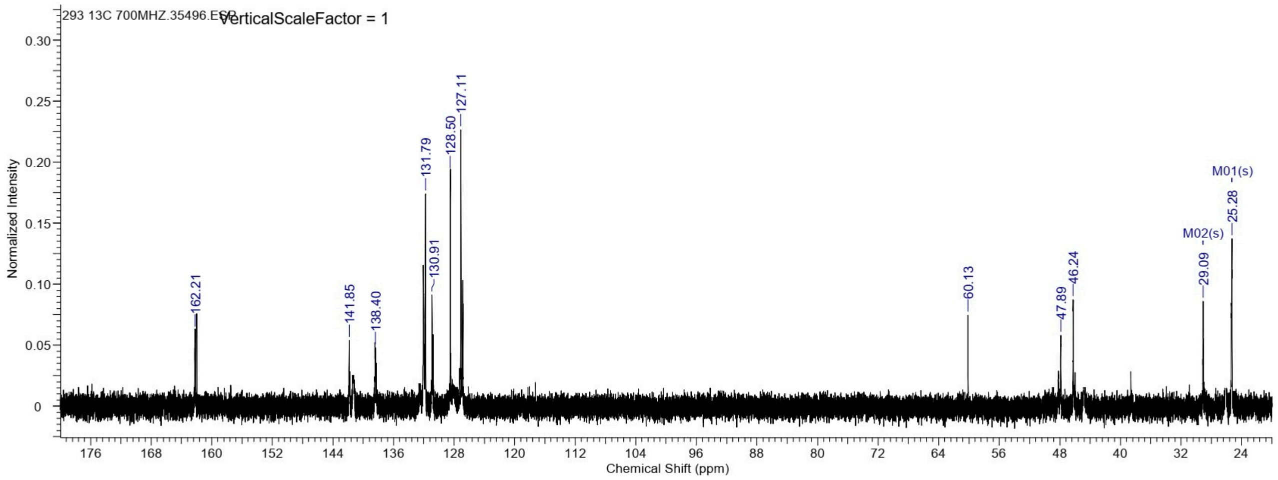

ESI-

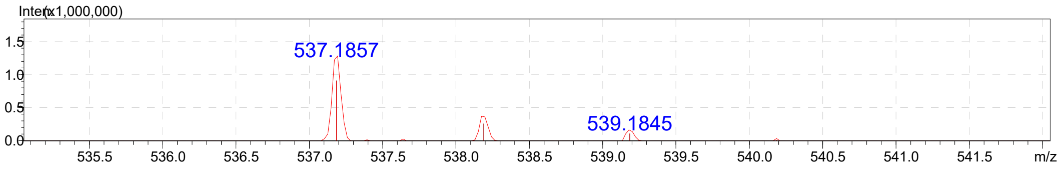

Sodium 2-((E)-((3-((4-(((E)-2-sulfonatobenzylidene)amino)butyl)amino)propyl)imino)methyl) benzenesulfonate (16)

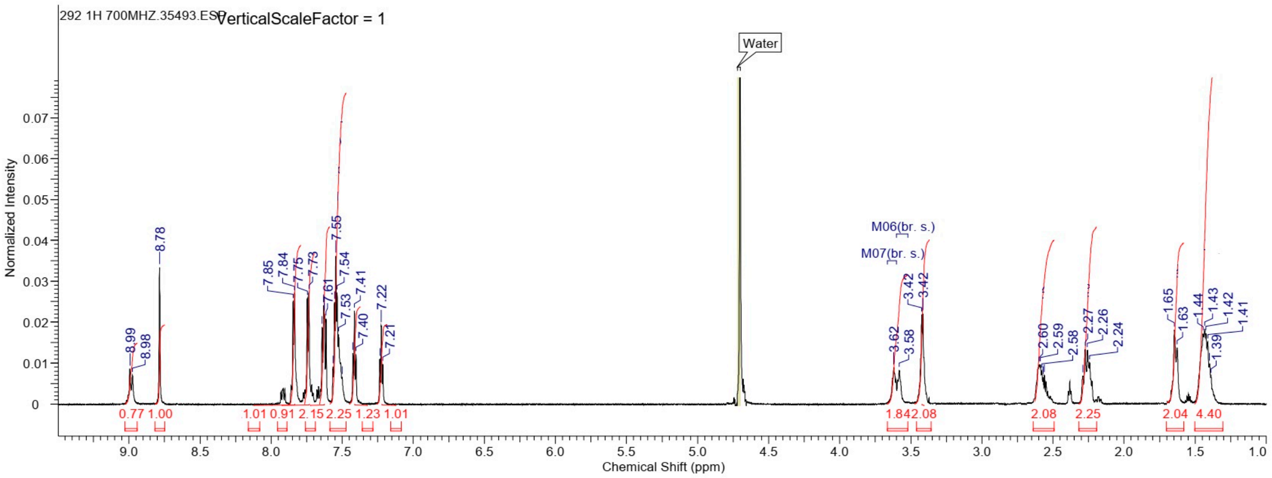

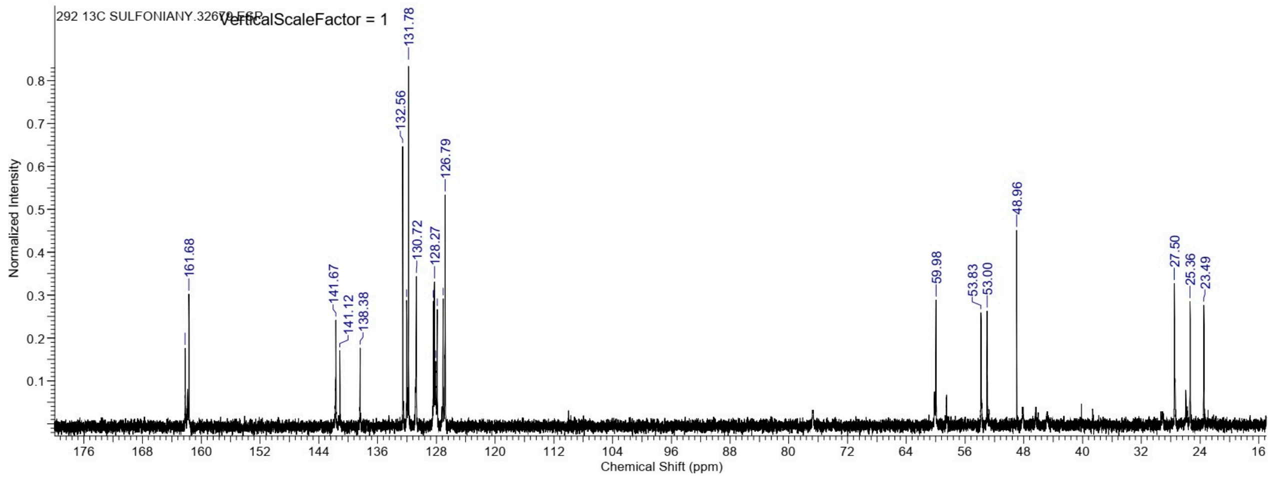

ESI+

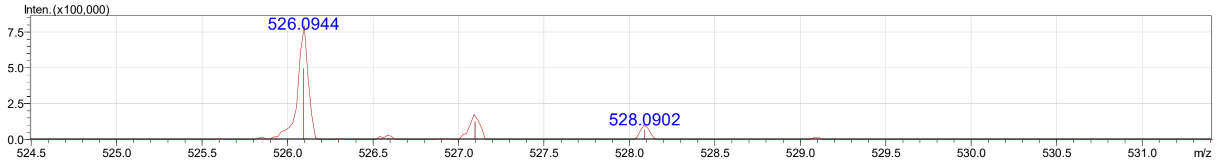

Supplement: Supplementary file 1 [file materials-17-00520-s001.zip › materials-2809743-supplementary.pdf]
